# Advanced drug delivery and therapeutic strategies for tuberculosis treatment

**DOI:** 10.1186/s12951-023-02156-y

**Published:** 2023-11-09

**Authors:** Ayushi Nair, Alosh Greeny, Amritasree Nandan, Ranjay Kumar Sah, Anju Jose, Sathish Dyawanapelly, Vijayabhaskarreddy Junnuthula, Athira K. V., Prashant Sadanandan

**Affiliations:** 1 Department of Pharmaceutics, Amrita School of Pharmacy, Amrita Vishwa Vidyapeetham, AIMS Health Sciences Campus, Kochi, 682 041 Kerala India; 2 Amrita School of Pharmacy, Amrita Vishwa Vidyapeetham, AIMS Health Sciences Campus, Kochi, 682 041 Kerala India; 3 Department of Pharmacology, Amrita School of Pharmacy, Amrita Vishwa Vidyapeetham, AIMS Health Sciences Campus, Kochi, 682 041 Kerala India; 4 Department of Pharmaceutical Sciences and Technology, Institute of Chemical Technology, Mumbai, 400019 India; 5 Drug Research Program, Faculty of Pharmacy, University of Helsinki, Viikinkaari 5 E, 00790 Helsinki, Finland; 6 Department of Pharmaceutical Chemistry, Amrita School of Pharmacy, Amrita Vishwa Vidyapeetham, AIMS Health Sciences Campus, Kochi, 682 041 Kerala India

**Keywords:** Drug delivery systems, Extensive drug-resistant tuberculosis, Multidrug-resistant tuberculosis, Nanoparticles, Therapeutics, Tuberculosis

## Abstract

Tuberculosis (TB) remains a significant global health challenge, necessitating innovative approaches for effective treatment. Conventional TB therapy encounters several limitations, including extended treatment duration, drug resistance, patient noncompliance, poor bioavailability, and suboptimal targeting. Advanced drug delivery strategies have emerged as a promising approach to address these challenges. They have the potential to enhance therapeutic outcomes and improve TB patient compliance by providing benefits such as multiple drug encapsulation, sustained release, targeted delivery, reduced dosing frequency, and minimal side effects. This review examines the current landscape of drug delivery strategies for effective TB management, specifically highlighting lipid nanoparticles, polymer nanoparticles, inorganic nanoparticles, emulsion-based systems, carbon nanotubes, graphene, and hydrogels as promising approaches. Furthermore, emerging therapeutic strategies like targeted therapy, long-acting therapeutics, extrapulmonary therapy, phototherapy, and immunotherapy are emphasized. The review also discusses the future trajectory and challenges of developing drug delivery systems for TB. In conclusion, nanomedicine has made substantial progress in addressing the challenges posed by conventional TB drugs. Moreover, by harnessing the unique targeting abilities, extended duration of action, and specificity of advanced therapeutics, innovative solutions are offered that have the potential to revolutionize TB therapy, thereby enhancing treatment outcomes and patient compliance.

**Graphical Abstract:**

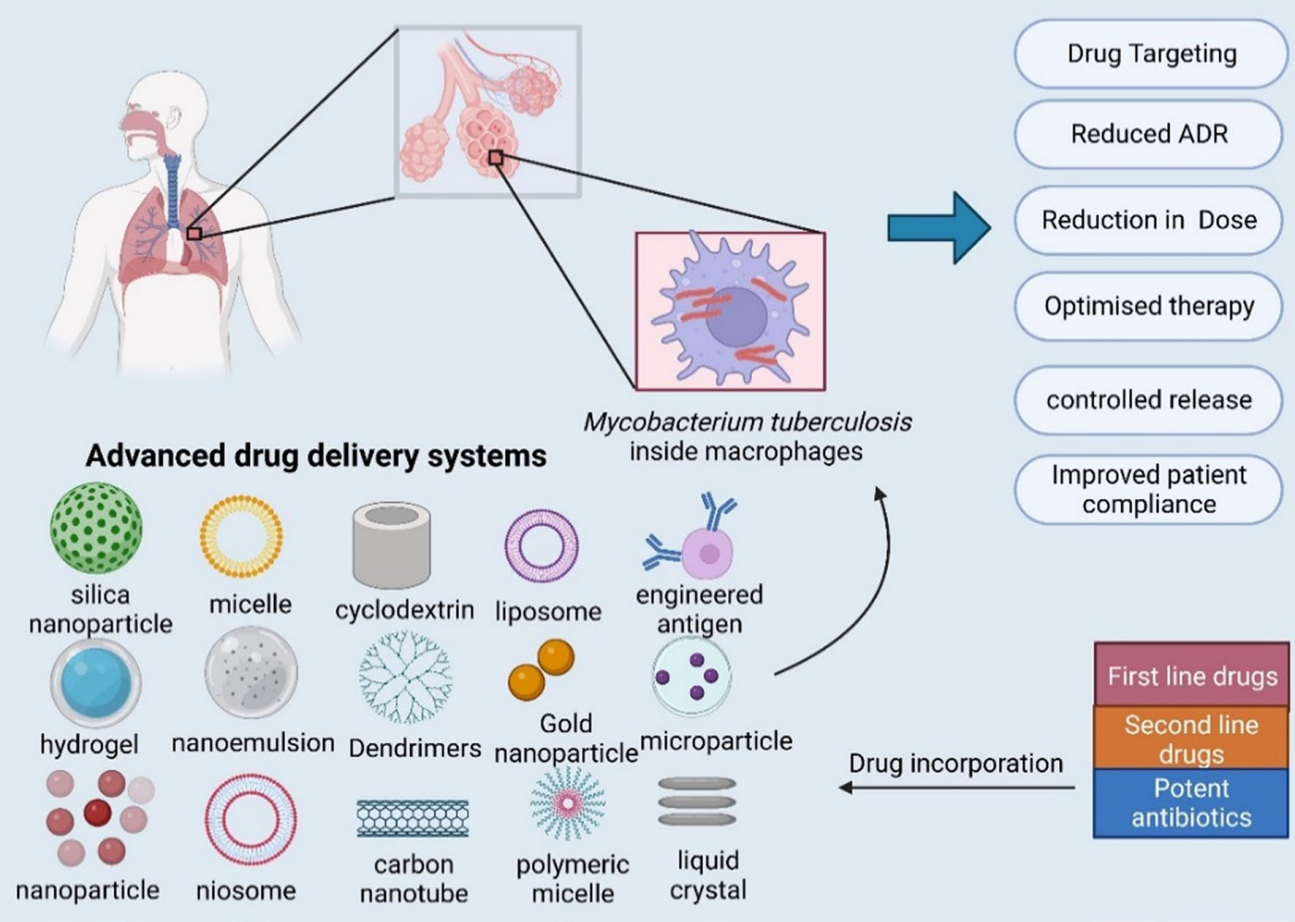

## Introduction

Tuberculosis (TB), a chronic granulomatous disease caused by *Mycobacterium tuberculosis* (*M. tuberculosis*) that typically infects the lungs, is one of the most prevalent contagious infections [1]. This aerosol-based transmissible disease is among the top infectious diseases worldwide [2–4]. In India alone, 26% of global cases have been reported, the most significant number of TB cases for any individual country [5]. It is considered the second deadliest infection after COVID-19. This infection is prevalent in all age groups worldwide and is curable as well as preventable. According to the WHO, early diagnosis and proper treatment have saved more than 74 million lives in the past two decades [6]. When the infection is untreated, the TB bacteria multiply and progress to other organs, which can result in fatal outcomes. Many patients diagnosed with TB are prescribed a standardized treatment regimen containing first-line anti-tubercular drugs (ATDs). The nonadherence of patients to ATDs leads to the generation of a newer strain known as multidrug resistant (MDR)-TB [7]. This occurs due to random chromosomal mutations and genetic changes in the bacterium. MDR bacilli are resistant to two important first-line drugs, isoniazid (INH) and rifampicin (RIF), and are treated using second-line ATDs, such as amikacin, capreomycin, and fluoroquinolones. Extensively drug-resistant (XDR)-TB is a more dangerous strain than MDR-TB. The treatment of XDR-TB is more difficult, as patients are resistant to many of the second-line drugs. Highly potent antibiotics, such as thioridazine, have severe side effects and are used for the treatment of XDR-TB [8].

**Fig. 1:**
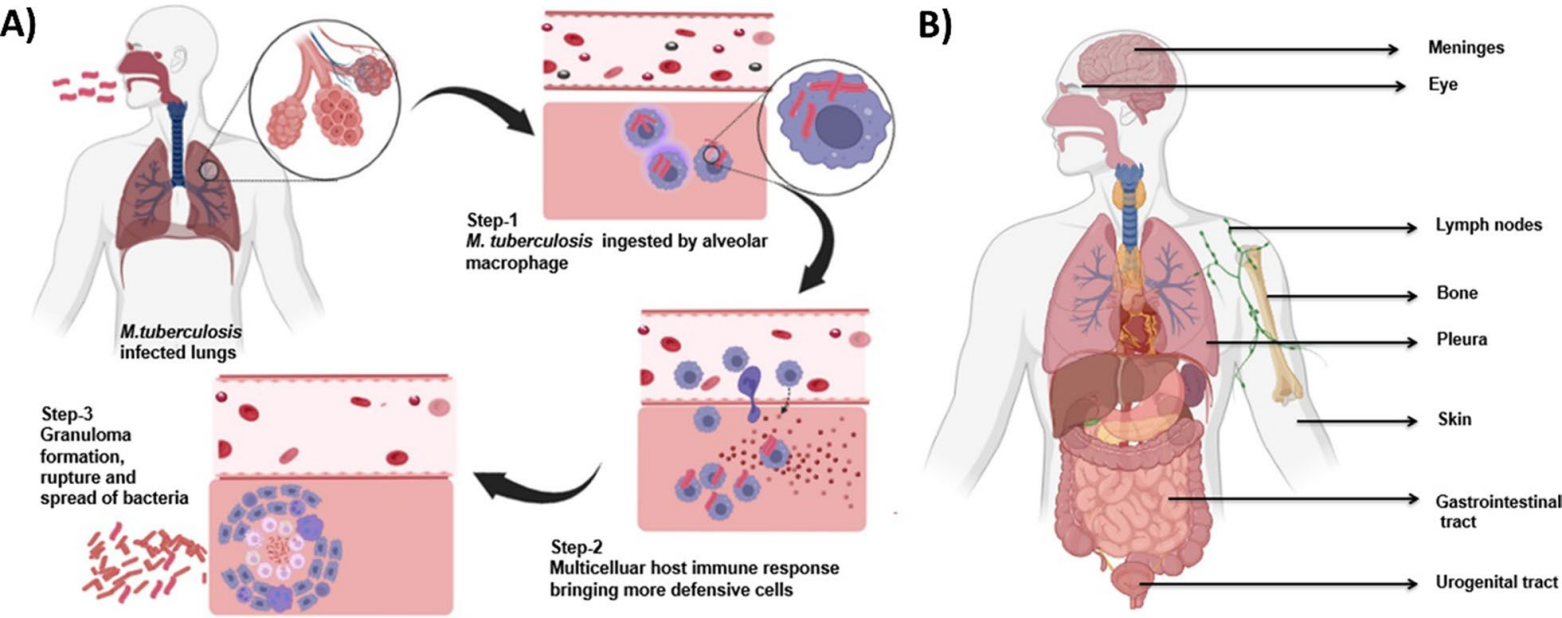
Pathogenesis of TB infection. **A** Pulmonary TB: by inhalation of infected droplet nuclei, *M. tuberculosis* enters the respiratory tract and alveoli of the lungs. It is ingested by AM, which attempts to destroy the bacilli initially. The development of symbiosis results in logarithmic growth of bacilli. The multicellular host immune response develops, bringing more defensive cells to the site. The infected areas progress into a granuloma that can develop a solid caseous center, where the bacteria survive for years, resulting in latent TB. In the final stage of pathogenesis, liquefaction of the caseous center and granuloma rupture cause the spread of bacilli. Pulmonary TB is caused by bacterial spread in the lungs. Extrapulmonary TB results from the dissemination of bacilli to other tissues and organs via the vascular or lymphatic system. **B** Extrapulmonary TB; common sites include the pleura, lymph nodes, gastrointestinal tract, urogenital tract, skin, bones, meninges or eye [14, 15]

Due to their size, the tubercle bacilli can reach the pulmonary alveoli, further becoming phagocytized by the alveolar macrophages (AM) [9, 10]. The bacilli then multiply in the alveolar sacs. The granulomas formed in these regions have heterogeneous size distributions and varying cellular compositions. When the bacterial load reaches a maximum, it can alter the morphology of granulomas, eventually spreading to other organs through the bloodstream and lymphatic system, resulting in extrapulmonary TB [11]. Thus, apart from the most common form of pulmonary TB, it can practically affect all human body organs, particularly the pleura, lymph nodes, abdomen, genitourinary tract, skin, meninges, joints, and bones [12, 13]. A schematic diagram of the pathogenesis of TB infection is shown in Fig. 1.

The treatment of TB patients is performed by using the TB-DOTS (directly observed treatment, short-course) treatment regimen. This regimen involves the use of various ATDs. The majority of the drugs used for therapy have severe adverse effects. The drugs' cure rates are as high as 95% in clinical trials, but they perform significantly worse in clinical conditions. The main reason for this is the lengthy duration of treatment and high dropout rates. The long duration of treatments and the serious adverse effects of the drugs impair patients' physical and mental endurance throughout therapy [16, 17]. Such instances cause patient relapse while also contributing to the development of bacterial resistance. Furthermore, there are several TB subpopulations, each with its physiology within the host. It can exist in two states that react to drugs differently: an active dividing state and a dormant/inactive state. It can alter drug metabolism, which also affects how effectively the therapy cycle functions [18, 19].

Due to tremendous improvements in treatment strategies, the number of patients infected with this disease is declining. The main reason for such a decline is the early and accurate diagnosis of the disease by various conventional and advanced techniques, such as chest X-ray, sputum microscopy, culturing method, nucleic acid amplification, ultralow dose chest CT (Clinical trials.gov identifier: NCT03220464), QuantiFERON (Clinical trials.gov identifier: NCT00982969), Nanodisk MS assay (Clinical trials.gov identifier: NCT03271567), and automated molecular diagnosis platform (Clinical trials.gov identifier: NCT04988984). However, due to the emergence of variant strains, the eradication of TB has not been achievable [20]. In this context, improved treatments with appropriate routes of administration are needed to shorten TB treatment duration, enhance efficacy, reduce adverse effects, and prevent resistance [9].

The significant challenges of clinical efficacy for TB include drug-resistant strains of *M. tuberculosis*, standard treatment duration (6–9 months), delayed diagnosis, and reduced therapeutic response with immunocompromised patients, which leads to more severe disease and a higher risk of complications that affect clinical outcomes [12, 21]. TB therapy is often delivered through various routes to ensure effective treatment. The main challenges associated with the oral route include slower onset of action, hepatic first-pass metabolism and rapid gastrointestinal absorption [9]. The parenteral and pulmonary routes for TB therapy displayed the highest bioavailability compared to oral administration. In particular, inhaled formulations are considered suitable for improving the pharmacodynamic profile of a drug [22]. Remarkably, lower doses of TB drugs can be delivered through inhalation and still result in effective treatments, thus reducing the chance of toxicity and enhancing localized drug concentrations [23]. With nanotechnology projected to simplify dosing and minimize adverse events, it can contribute significantly to the elimination of TB, especially by eradication of mycobacteria in those who do not have an active disease (latent TB), thereby preventing the progression to active disease [14, 24].

Advanced drug delivery strategies could improve bioavailability, patient compliance and the efficacy of TB treatment. As summarized in Table 1, with the dearth of new drug approvals for ATDs, except for bedaquiline, delamanidin, and pretomanid, which have come up in more than 40 years, innovative drug delivery strategies for existing drugs could be considered promising to enhance patient compliance [25–28]. Novel drug delivery systems (NDDSs) aid in optimizing the concentration of the active compound in the patient's plasma. Promising strategies for optimizing drug delivery could be based on modern systems such as nanoparticles, liposomes, microemulsions, niosomes, dendrimers and liquid crystalline systems [3, 25, 29]. By developing inexpensive and easy-to-administer delivery systems that offer extended drug release, dosing frequency could be reduced, thereby improving patient adherence. Direct targeting by selectivity toward both AM and tubercle bacilli may counteract the ability of intracellular pathogens to evade antibiotic treatments [30]. Thus, NDDSs can help optimize drug delivery to the target site, maximizing drug absorption and minimizing unwanted side effects [31]. Moreover, drug delivery systems can be optimized for a suitable route of administration to safeguard the therapeutic agents from immediate host metabolism and clearance, which can aid in therapeutic dose reduction [32]. A well-designed drug carrier can also demonstrate controlled drug release characteristics as per different metabolic and physicochemical responses [33]. These delivery systems also have a high possibility of treating nontubercular infections in the future [34, 35].

**Table 1:** Drugs used in tuberculosis therapy

Drug classification	Drugs	Route of administration	Dosage form	Half-life (Hours)	Bioavailability (Percentage)	References
First-line drugs	Isoniazid	Oral	Tablet	1–4 h	78 – 93%	[16]
	Rifampicin	Oral	Tablet	2–5 h	71–87%	[36]
	Pyrazinamide	Oral	Tablet	9–10 h	> 90%	[37]
	Ethambutol	Oral	Tablet	3–4 h	82–87%	[38]
Second-line drugs	Streptomycin	*i.m*	Powder for solution for injection	2– 4 h	84–88%	[39]
	Amikacin	*i.m.*, *i.v*	Solution for injection, powder for injection	2–3 h	95.2%	[40]
	Kanamycin	*i.m.*, *i.v*	Solution for injection, powder for injection	4–6 h	very low	[41]
	Ofloxacin	Oral, *i.v*	Tablet, infusion	5–8 h	85–95%	[42]
	Levofloxacin	Oral, *i.v*	Tablet, powder for injection, oral solution	4–7 h	80–100%	[43]
	Moxifloxacin	Oral, *i.v*	Tablet, infusion	12–14 h	90%	[44]
	Ciprofloxacin	Oral, *i.v*	Tablet, infusion	4 h	70–80%	[45, 46]
	Clarithromycin	Oral	Tablet, granules for oral suspension	2 h	55%	[47]
	Cycloserine	Oral	Capsule	16–20 h	80%	[48]
	Para-amino salicylic acid	Oral	Delayed-release granules	1 h	20%	[49]
	Clofazimine	Oral	Soft-gel capsule	10 days	68 – 95%	[50]
	Capreomycin	*i.m.*, *i.v*	Powder for injection	4.8 ± 1 h	59%	[51]
	Ethionamide	Oral	Tablet	2–3 h	90–100%	[52]
	Prothionamide	Oral	Tablet	1.5–2 h	33%	[53, 54]
	Terizidone	Oral	Capsule	5.27–17.8 h	100%	[55, 56]
	Rifabutin	Oral	Tablet	> 30 h	20%	[57]
	Linezolid	oral, *i.v*	Tablet, infusion	5–7 h	100%	[58]
	Bedaquiline	Oral	Tablet	164 days	Unknown	[59]
	Pretomanid	Oral	Tablet	~ 17 h	< 50%	[60]

This table includes all the first-line and second-line ATDs and their pharmacokinetic parameters*.* As per WHO guidelines, the standard 6-month regimen for the treatment of drug-susceptible TB includes daily administration of isoniazid (H), rifampicin (R), pyrazinamide (Z), and ethambutol (E) for two months, followed by daily administration of isoniazid and rifampicin for an additional four months; 2HRZE/4HR. There is a new recommendation for the treatment of people aged 12 years or older with drug-susceptible pulmonary TB that they may receive a 4 month regimen of isoniazid (H), rifapentine (P), moxifloxacin (M), and pyrazinamide (Z); 2HPMZ/2HPM. Second-line drugs are used when treatment with first-line drugs fails or in the presence of multidrug-resistant TB (MDR-TB). Levofloxacin, moxifloxacin, clofazimine, linezolid, bedaquiline and pretomanid are the prominent drugs under drug-resistant TB treatment regimens. *i.m*. intramuscular, *i.v*. intravenous

Here, we have reviewed the recent advances in drug delivery and therapeutics to effectively treat TB by overcoming the existing hurdles associated with TB therapy. We have discussed promising preclinical developments and their future perspectives, along with the challenges that must be addressed for impactful clinical translation.

## Advanced drug delivery strategies

**Fig. 2:**
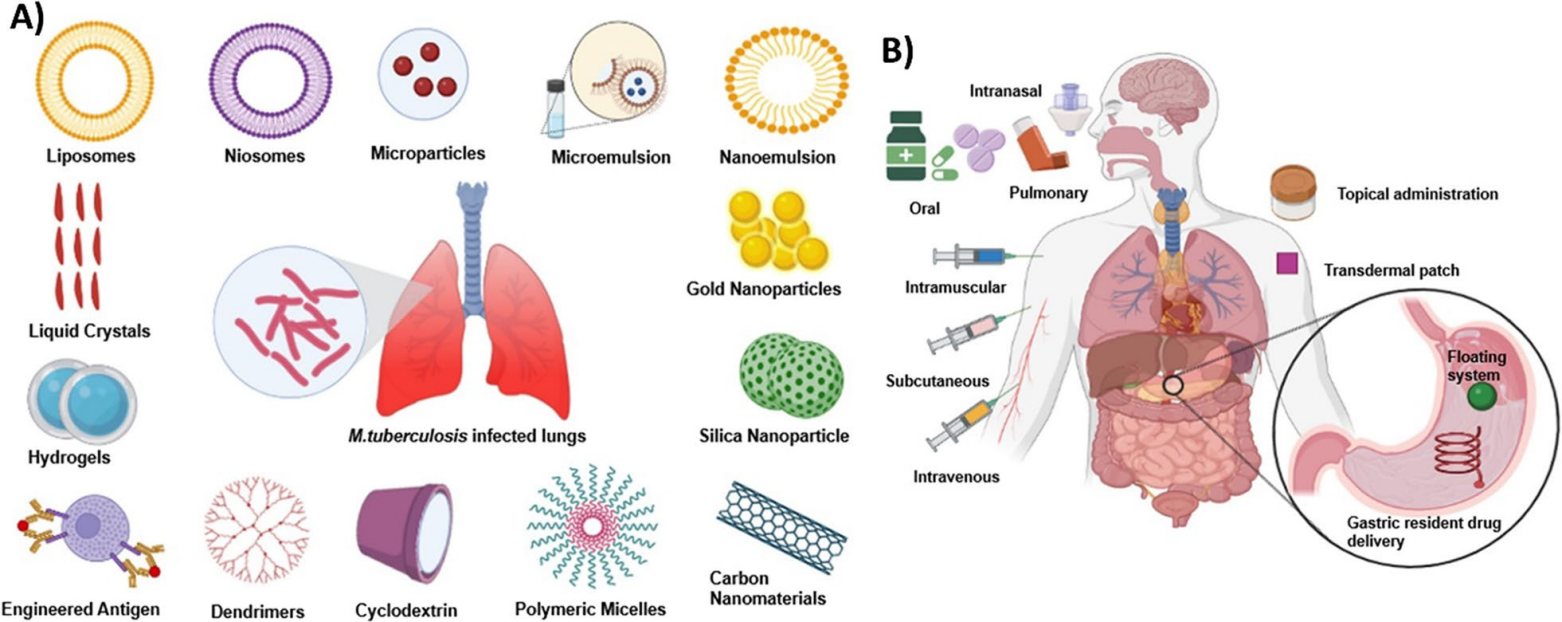
Modern drug delivery systems for bioactive molecules in TB treatment. Modern drug delivery strategies for existing TB drugs could be promising, as they can offer the flexibility to adopt better routes of administration, multiple drug encapsulation, sustained drug release, targeted drug delivery, enhanced permeability and retention along with a lower incidence of side effects. **A** Promising strategies for optimizing drug delivery could be based on liposomes, niosomes, microparticles, microemulsions, nanoemulsions, gold nanoparticles, silica nanoparticles, carbon nanomaterials, polymeric micelles, CD, dendrimers, engineered antigens, hydrogels, and liquid crystals. **B** The promising preclinical developments for TB treatment involve varying routes of administration, such as oral, intranasal, pulmonary, topical, intramuscular, intravenous and subcutaneous routes. Newer approaches include transdermal patches, floating systems, and a one-time large-dose controlled-release gastrointestinal resident delivery system

Designing NDDSs includes approaches for achieving and optimizing the continuous delivery of drugs in a precise and reproducible manner to the target site. A major focus has been on targeted drug delivery and minimizing undesirable effects. They are of various types depending upon the formulation, dosage form, and mechanism of drug delivery. Various types of delivery systems and routes of administration for TB are represented in Fig. 2.

Nanocarriers offer prominent advantages, such as drug release in the presence of specific triggers, providing temporal control of drug exposure, enhanced drug uptake in target cells, improved efficacy against intracellular pathogens, and protection of labile therapeutic agents from harsh physiological conditions such as low pH or enzymatic degradation [61]. In particular, polymers such as poly(lactic-co-glycolic acid) (PLGA), poly(caprolactone), poly(anhydrides), poly(orthoesters), poly(cyanoacrylates), and poly(amides) have opened avenues to modify drug release patterns by altering the monomer hydrophobicity, polymer chain length and particle size [62]. However, encapsulation of both hydrophobic and hydrophilic drugs is possible with liposomes to achieve sustained release [14].

Recent advances in nanotechnology have brought carriers into the limelight, which can specifically target AM [30]. Modulation of the physical properties of drug carriers, such as surface composition, charge, shape, particle size, hydrophobicity, and zeta potential, can modify drug internalization by AM. Another approach has been to use ligands on the nanocarrier that interact with specific receptors on macrophages, termed active targeting or ligand-mediated targeting. Strategies that do not rely on specific ligands have been called passive targeting. For passive targeting of AM, polymer- and polysaccharide-based carriers, as well as liposomes, solid lipid nanoparticles (SLNs), and gold nanorods, have been utilized for numerous agents, mostly INH and RIF [30]. Polysaccharides and their derivatives, including chitosan, inulin, alginate, and CD, have seen increased applications owing to their biocompatibility, biodegradability, hydrophilicity, mucoadhesive properties, use in modifying carrier surface charge, and target specificity [63–66].

Despite the beneficial prospects of these modern and fabricated drug delivery systems, safety and toxicity need to be verified, as they comprise different types of material [67, 68]. These technological solutions need to support scalability and reproducibility, aiding clinical translation beyond laboratory optimization by overcoming manufacturing, regulatory and financial challenges [69]. In particular, the reformulation of existing drugs for enhanced efficacy and safety needs to be cost-effective [70]. Furthermore, the synthesis and storage conditions need to be conducive to conditions in low-resource countries [71]. Importantly, process optimization is of utmost significance in the case of nanomedicines that are likely to be 3D constructs of multiple components with preferred spatial arrangements, with any deviation adversely affecting the composition [72, 73]. Table 2 provides a summary of drug delivery systems and their key findings incorporating anti-tubercular drugs.

**Table 2:** Drug delivery systems for anti-tubercular drugs

Drug	Delivery system	Key findings	References
First line anti tubercular drugs			
Isoniazid	Liposome	Dual purpose of pulmonary drug delivery and alveolar stabilization due to antiatelectatic effect of the surfactant action	[74]
	Niosome	Due to the targetability of the drug a low dose of the drug can provide efficient treatment of TB	[75]
		Optimum level of drug entrapment efficiency, reducing the dose, dosing frequency, and toxicity in J744A.1 mouse macrophages	[76]
	Aluminum nitride- and aluminum phosphide-doped graphene quantum dots	Less toxic and more hydrophobic	[77]
	Chitosan nanotube	Prolonged the release time of the drug, providing a uniform release rate	[78]
	Multiwall carbon nanotubes	Increased lethality against *M. tuberculosis*	[79]
	Mannitol microsphere containing iron (III) trimesate metal–organic framework MIL-100 nanoparticles	↑ Encapsulation efficiency and aerodynamics; efficient internalization in cytoplasm, making it suitable for deep lung delivery	[80]
	Hydrogel-forming microneedle arrays	↑ Permeation aiding transdermal delivery with lyophilized reservoir	[81]
	Calcium ion-Sodium Alginate-Piperine-based microspheres	↑ Entrapment efficiency; prolonged release and oral bioavailability	[82]
Rifampicin	Niosome	By controlling the niosome size, major portion of the drug can be concentrated in the lung region	[83]
		Effective compartmentalization of the drug can be achieved in the lymphatic system	[84]
	Mannosylated dendrimer	↓ Drug release rate in pH 7.4; ↑ Drug release in pH 5.0 and alveolar macrophage uptake; biocompatibility; site-specific delivery	[85]
	Microsphere	Preferential accumulation of drug in lungs; delivery can be done through respiratory tract	[86]
	Liposome	Drug release in a controlled manner for a longer period of time	[87]
	G4-PAMAM dendrimer	Higher stability and pH depended release of the drug	[88]
	Liquid-crystalline folate nanoparticle	Sustained release; ↓ Cytotoxicity	[89]
	G1-G3 PAMAM dendritic microsphere	PAMAM G3 dendritic microsphere was identified as the suitable drug carrier for the pulmonary delivery	[90]
	Liquid crystalline nanoparticles	↓ Minimum inhibitory concentration (MIC) against *S*.*aureus* due to enhanced solubility and strong membrane fusion of drug	[91]
	Mono-oleate based liquid crystals	Sustained release and 93% loading frequency	[92]
	Alginate-cellulose nanocrystal hybrid nanoparticles	↑ Drug encapsulation and sustained release action	[93]
	Inulin functionalized with vitamin E (INVITE) micelle	↑ Mucoadhesion properties to the mucin and comparable antimicrobial property against gram-positive bacteria	[94]
	Cross-linked poly-β-cyclodextrin (p-β-CD) nanoparticles	Direct lung targeted delivery; pβCD nanoparticles on their own or loaded with antibiotics have anti-TB action	[95]
	Polymeric micelles	deep lung drug delivery	[96]
	Mannosylated and PEGylated graphene oxide carrier system	Selective macrophage targeting; ↑ Intracellular drug concentration	[97]
	Nanoemulsion	Effective ophthalmic drug delivery; Electrostatic interaction with mucin leading to increased residence time	[98]
	Hydrogel-forming microneedle arrays	↑ Permeation aiding transdermal delivery on combination with poly(ethylene glycol)	[81]
Pyrazinamide	Hydrogel-forming microneedle arrays	↑ Permeation aiding transdermal delivery with lyophilized reservoir	[81]
Ethambutol	Niosome	↑ Lung targeting; superior biological as compared to free drug	[99]
	Solid lipid nanoparticles	Targeted drug delivery; ↓ Dosing frequency; ↑ Bioavailability	[100]
	Hydrogel-forming microneedle arrays	↑ Permeation aiding transdermal delivery on combination with directly compressed tablet	[81]
Second line anti tubercular drugs			
Streptomycin	Liposome	↓ In the number of mycobacteria in spleen, but not in lungs	[101]
Amikacin	Liposome	↓ Viable bacterial count in the liver and spleen	[102]
Levofloxacin	Liposome	↓ MIC	[103]
Rifabutin	Liposome	↓ Lung pathology; ↓ Bacterial load in the spleen and liver	[104]
		↑ Activity against *M. avium*	[105]
Clofazimine	Liposome	↑ Half-life and biodistribution	[106]
		↓ Bacterial load in the liver, spleen and kidneys	[107]
		↓ Viable bacterial count in lung, liver and spleen at all infection levels	[108]
		↓ Bacterial load in spleen, liver and lungs	[109]
	Cyclodextrins	β-CD showed the best inclusion capacity, sufficient pulmonary bioavailability and in vitro deposition performance in lungs	[110]
Para-amino salicylic acid	Graphene oxide air-dried hydrogel	Strong antibacterial activity; more invasive	[7]
Moxifloxacin	Poly(butyl cyanoacrylate) nanoparticles	Distribution of nanoparticles near the vicinity of the bacteria	[111]
Ethionamide	Biodegradable polymeric nanoparticles	Simultaneous delivery of ethionamide and its booster BDM41906 in "green" β-CD-based nanoparticles showed the best physico-chemical characteristics; ↓ Pulmonary mycobacterial load	[112]
	Spray-dried microparticles	↑ Absorption; higher AUC_(0-t)_; ↑ Bioavailability	[113]
Linezolid	Graphene oxide	↑ Bactericidal activity	[114]

### Lipid-based drug delivery systems

#### Liposomes

The efficiency of drug delivery by liposomal systems has mainly been studied using *M. avium* and *M. tuberculosis* models. In a mouse model, liposomal rifabutin was demonstrated to slow the pathogenic course of TB infection. Formulations containing dipalmitoyl phosphatidylcholine and dipalmitoyl phosphatidylglycerol were able to reduce TB progression in the lungs and lowered the bacterial loads in the spleen and liver, implying that liposomal-loaded ATDs could be a promising approach for treating extrapulmonary TB [104]. Similarly, liposomal clofazimine showed higher antibacterial activity than free clofazimine against the *M. avium* complex in mice, even when the treatment was initiated after the dissemination of the infection [108].

RIF and INH-encapsulated liposomes reduced pulmonary inflammation and enhanced the survival of TB-induced mice. Liposome formulation improved RIF penetration across the alveolar epithelium, extending pulmonary residence time and lowering systemic drug toxicity [116]. Mannan-anchored liposomes containing RIF, INH, and pyrazinamide can be delivered to the lungs using a dry powder inhaler (DPI) for the treatment of pulmonary TB with high entrapment efficiency and sustained drug release [117].

The liposome-in-hydrogel technique was found to be promising for treating bone TB locally. Liposomes entrapped with INH served dual functions of pulmonary medication transport and alveolar stabilization. DPIs containing ligand-anchored pH-sensitive liposomes for the simultaneous delivery of INH and ciprofloxacin demonstrated the greatest accumulation in the lung. Liposomes can aid in the pulmonary administration of ATDs, which could be an attractive alternative to improve TB therapy [74, 118, 119].

Asymmetric liposomes made up of phosphatidylserine at the outer surface resembling apoptotic bodies and phosphatidic acid at the inner layer could be used to boost innate antimycobacterial activity in phagocytes while limiting the inflammatory response [120]. Liposomes containing dipalmitoyl phosphatidylcholine and cholesterol were reported to inactivate *M. tuberculosis* and multidrug-resistant (MDR) -*M. tuberculosis*, with action dependent on the incubation period and low dose [121]. Phosphatidylcholine-cholesterol-cardiolipin liposome formulation and levofloxacin are efficacious against *M. tuberculosis* bacilli [103]. Liposomes may potentially be useful as part of a gene vaccine for TB treatment, as evidenced by the efficacy of a peptide-DNA-cationic liposome pseudoternary complex [122].

#### Niosomes

INH integrated with niosomes was found to be effective against TB, with 62% cellular absorption by macrophages and 65% drug localization to the target organ, compared to 15% with the supplied free INH [123]. Liposomal preparations of azole antifungals such as clotrimazole and econazole were shown to be effective against *M. tuberculosis* and latent bacilli, while fluoroquinolones such as moxifloxacin accumulated more efficiently in AM when delivered in the context of niosomes [123].

Chowdhury et al. [208] and El-Ridy et al. investigated niosomal formulations of RIF and ofloxacin for the treatment of drug-resistant TB, finding 81.76% entrapment efficiency and regulated release for up to 15 days. El-Ridy et al. formulated ethambutol-containing niosomes that demonstrated regulated release and a reduction in nonspecific toxicity [99]. After intratracheal injection, RIF-loaded niosomes demonstrated enhanced accumulation and 90% RIF release in 48 h, with 65% localization [125]. Niosomes containing cholesterol and Triton X-100 successfully targeted RIF in Wistar rats, INH in J744A.1 mouse macrophages, and ethambutol in Swiss albino mice infected with *M. tuberculosis* H37Rv [76, 126]. In guinea pigs, pyrazinamide encapsulated in niosomes demonstrated significant drug entrapment efficiency. Subcutaneous treatment with niosomal ethambutol formulated with Span 60, Span 85, cholesterol, diacetyl phosphate, and stearyl amine resulted in prolonged drug release in Swiss albino mouse lungs and lowered bacterial counts in guinea pigs infected with *M. tuberculosis* H37Rv by i.m. injection [99, 127].

For highly lipophilic drugs, such as BM859, which demonstrated significant antimycobacterial action against *M. tuberculosis* H37Rv, niosomes are a preferable method of drug delivery. Sadhu et al*.* developed ethionamide niosomes with sufficient stability for intravenous injection [128, 129]. Niosomes loaded with hydrophilic D-cycloserine and lipophilic ethionamide kill drug-resistant TB by releasing 96% of ethionamide and 97% of D-cycloserine [130]. Antimycobacterial drugs encapsulated in tyloxapol niosomes have a high drug loading efficiency, and the isatin-INH hybrid WF-208 has a fourfold higher MIC against H37Rv *M. tuberculosis* [131–133].

#### Liquid crystals

**Fig. 3:**
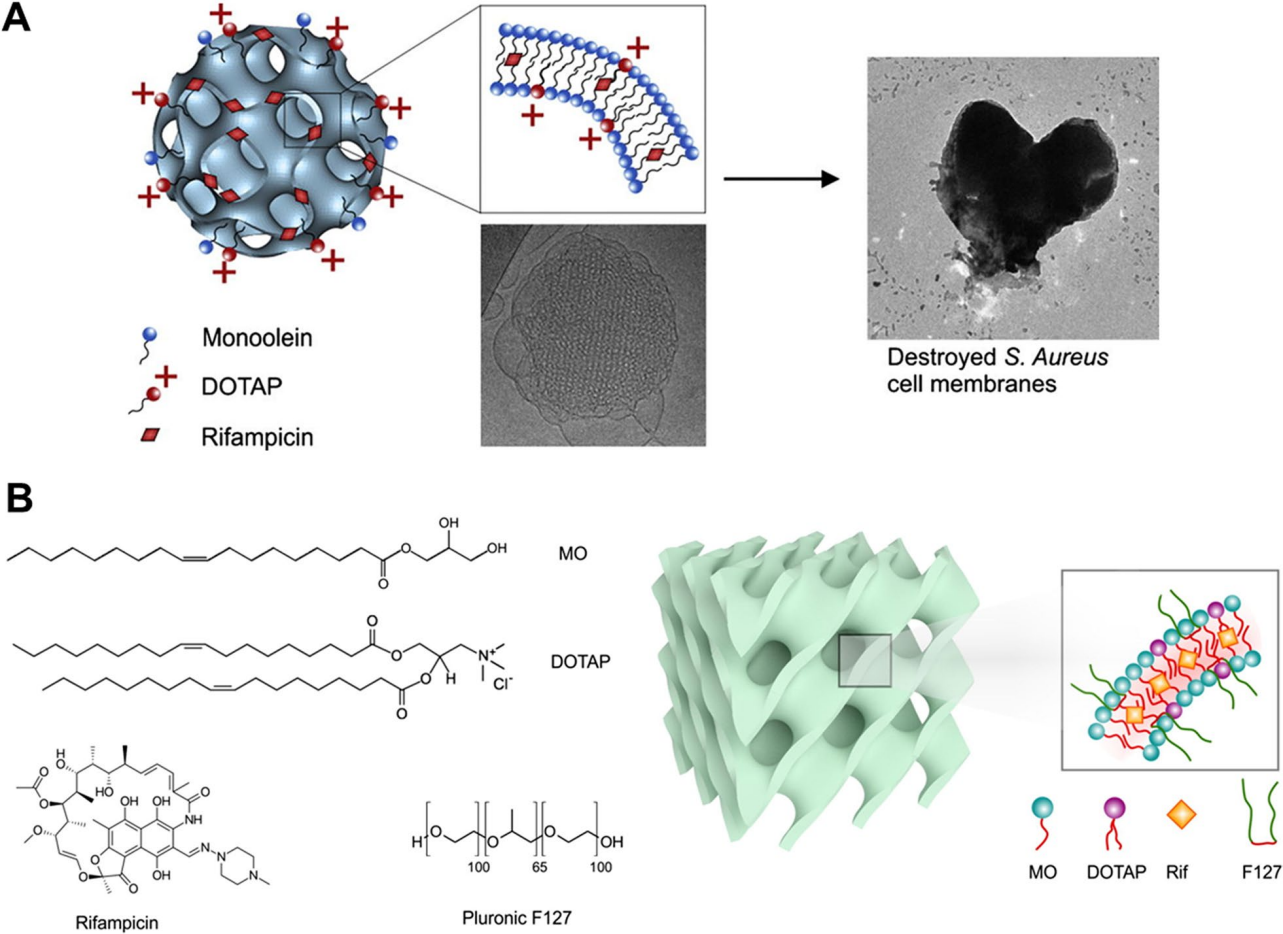
Schematic representation of nonlamellar lyotropic liquid crystalline nanoparticles. **A** Cationic charge effect on the *S. aureus* cell membrane. **B** Chemical structures of monoolein (MO), cationic lipid 1,2-dioleyl-3-trimethyl-ammonium-propane (DOTAP), antibiotic rifampicin (Rif), and Pluronic F127 modified and reprinted with permission from [91] © Elsevier Inc

Liquid crystals are an ideal mechanism of drug delivery for ATDs. Liquid crystal-based zidovudine, ciprofloxacin, and fluconazole formulations are some of the recent developments in the antimicrobial field, making them a possible approach for delivering ATDs as well [135]. K. Dua et al. synthesized RIF-based liquid crystals with higher solubility and stability in acidic environments than the free drug, allowing for lower dose frequency and increased bioavailability. RIF is poorly water soluble and degrades in the stomach, resulting in limited bioavailability [136]. Tran et al*.* created two RIF-loaded nanoparticles using neutral lipid monoolein and the cationic lipid N-(1-(2,3-Dioleoyloxy)propyl)-N,N,N-trimethylammonium methyl-sulfate (DOTAP), which decreased the MIC of RIF against *S. aureus*. The cationic charge helped in increased solubility and greater membrane fusion, as explained in Fig. 3 [91]. Kim et al*.* developed a liquid crystal-based aptasensor for IFN detection that has higher sensitivity and a lower detection limit than enzyme linked immunosorbent assay (ELISA). They created an improved biosensor that uses immobilized antigen ESAT-6 to detect anti-TB antibodies (anti-ESAT-6) [137]. Bedaquiline-loaded cubosomes based on nanocarriers have been demonstrated to be effective in the treatment of non-small cell lung cancer (NSCLC) by sustained release over 72 h [138].

### Polymer-based drug delivery systems

#### Polymer micelles

Because of their capacity to stabilize and protect the drug, prolong the therapeutic activity, and enclose hydrophobic pharmaceuticals, polymeric micelles are used as drug carriers. They can also be utilized to deliver multiple ATDs into infected macrophages while causing fewer negative effects [139, 140]. Tripodo et al*.* created RIF-delivering micelles based on inulin functionalized with vitamin E (INVITE) and its succinylated derivative (INVITESA). It demonstrated strong mucoadhesion to mucin and equivalent antibacterial activities against gram-positive bacteria [94].

RIF- and INH-loaded N-(2-hydroxypropyl)methacrylamide-poly(lactic acid) micelles allow prolonged drug release, improving effectiveness against resistant and sensitive pathogens. Kaur et al*.* revealed different polymeric micelle-based delivery methods for ATDs for drug-resistant TB, including polyethylene oxide-polypropylene oxide (PEO-PPO), polyvinyl-caprolactam-polyvinyl acetate-polyethylene glycol graft copolymer (PCL-PVAc-PEG), and chitosan-graft-poly-ε-caprolactone (CS-g-PCL). PEO-PPO decreased the MIC value, PCL-PVAc-PEG improved RIF solubility and physical stability, and CS-g-PCL provided INH with pH-dependent release, improved cellular internalization, and decreased cytotoxicity [141].

Yuan et al*.* created interconnected hydrogel micelles for the delayed release of weakly water-soluble RIF using biodegradable polymers such as guar gum, chitosan, and polycaprolactone. In vitro release experiments revealed that 90% of the medication was released in 12 days, and 97% of the encapsulation was effective [142]. Sheth et al*.* encapsulated RIF and INH in pluronic, which increased activity and sustained release against *M. tuberculosis*. Grotz et al*.* developed an inhalable nanocarrier based on RIF-loaded polymeric micelles to improve water solubility [96, 143].

#### Chitosan-based polymer drug delivery systems

Chitosan can be used as a vaccine delivery mechanism in the treatment of TB. Negatively charged particles, such as PLGA, can be coated with chitosan to transfer them efficiently to the mucosal membrane [144, 145]. Gu et al*.* recently proved that a combination of dihydroartemisinin and chitosan might overcome *M. tuberculosis* RIF resistance. This combination worked best at lower chitosan concentrations, but at greater concentrations, the bacteria were deprived of nutrition [146]. Chitosan biguanidine nanoparticles developed using a one-pot green synthesis method can be used as carrier systems for ATDs. It was demonstrated that chitosan biguanidine nanoparticles have enhanced pharmacological activity owing to targeted delivery [147].

#### Alginate-based polymer drug delivery systems

For MDR-TB, hydrophilic ATDs such as amikacin and moxifloxacin were encapsulated in alginate-entrapped PLGA nanoparticles. When these nanoparticles were fed to macrophages infected with *M. tuberculosis*, antibacterial activity was detected [148]. For the administration of RIF in combination with ascorbic acid, a nanocarrier system comprised of alginate coated with Tween 80 and chitosan was created [149]. Alginate-cellulose nanocrystal hybrid nanoparticles have demonstrated significant antimycobacterial action and moderate oral medication delivery difficulties [93]. Anti-TB action has also been shown in nanostructured polyelectrolyte complexes synthesized from sodium alginate and chitosan. Alginate particles have been investigated as a means of encapsulating live mycobacterium particles for use in inhalable vaccinations [150, 151].

Nagpal et al*.* proposed coating live mycobacterium with alginate to improve dendritic cell activation and maturation [150]. When alginate-coated chitosan nanoparticles were delivered intranasally and subcutaneously, they released the PPE17 antigen, which produced effective immune responses in mice [152]. Pregelatinized sodium alginate and chitosan can be used for the development of nanoparticles of INH and pyrazinamide and could be an interesting approach for TB treatment [153]. To improve stability and long-term release, polypeptidic micelles containing bedaquiline were coated with sodium alginate [154].

Zn-alginate beads show excellent biocompatibility and no fatal cytotoxicity when utilized as carriers for RIF administration [155]. Alginate has been employed as a stabilizer in the manufacture of silver nanoparticles, which have the ability to attack *M. tuberculosis* and sterilize nonreplicating persistent TB [156]. It has been discovered that calcium ion-sodium alginate-piperine-based microspheres improve entrapment efficiency and extend the release and oral bioavailability of INH [82].

#### Cyclodextrin-based polymer drug delivery systems

CDs are cyclic oligosaccharides with D-glucose units linked by β-1,4-glucosidic linkages [157]. Because of the constrained rotation around the bonds joining the glucopyranose units, they form a toroidal structure [158–160]. β-Cyclodextrins (β-CD) have the potential to be an effective carrier system for ATD delivery. An in vivo investigation found that administering unloaded β-CD via endotracheal or intranasal routes reduced the bacterial burden. Loaded with ethionamide and boosted with BDM43266, the bacilli activity was tenfold increased and selective. This can be used to combine several medications into a single formulation [95].

RIF is insoluble and permeable, but this can be addressed by creating an inclusion combination with hydroxypropyl- β -CD (HP- β -CD). To simplify dose modification and treatment adherence, Javier Suárez-González et al*.* developed a combined-dose oral pediatric formulation including INH and RIF. HP- β -CD has also been employed to create a powdered RIF dosage form for direct lung-focused distribution [95].

The bioavailability and deposition performance of clofazimine were increased by combining it with β-CD and L-leucine [110]. Curdlan nanoparticles containing RIF and levofloxacin were used to target *M. tuberculosis*-infected macrophages [161]. In mice, the combination of ethionamide with the booster BDM41906 decreases mycobacterial load [112]. Christian and Werner successfully complexed an INH-hydrazone-phthalocyanine compound in β-CD encapsulated in soybean lecithin liposomes. It demonstrated pH-dependent drug release that is appropriate for site-specific delivery [162]. Anjani et al*.* discovered that CD inclusion complexes improved antibacterial action, with 60% drug release in 2 h [139].

#### Dendrimer-based drug delivery systems

Polymers commonly used for the preparation of dendrimers are poly(amidoamine) (PAMAM) and poly(propylene imine) (PPI) [163–165]. Others include polyglycerol, poly(ether hydroxylamine) (PEHAM), poly(ester amine) (PEA), and melamine [166]. Dendrimers behave like unimolecular micelles that facilitate the delivery of both hydrophilic and hydrophobic drugs [167–169]. Cationic dendrimers can be used as nonviral gene carriers [170].

The RIF-PAMAM complex can be used as a carrier for drugs to acidic sites, as normal RIF can lead to solubility issues [171]. When a maximum of twenty RIF molecules were loaded in fourth-generation PAMAM dendrimers, a sustained release profile was observed at neutral pH, whereas simultaneous release was triggered at acidic pH. Incorporating RIF into G3 PAMAM prolonged its release compared to first- and second-generation PAMAM due to the entrapment of RIF in the branched chains of G3 PAMAM along with the high density, high molecular weight, and size of G3 PAMAM [173].

The solubilization of drugs is due to hydrophobic-hydrophilic interactions, the interaction between ions, and encapsulation of hydrophobic drugs into crevices of dendritic architecture, as demonstrated by Karthikeyan R et al*.* using known concentrations of PEGylated PPI dendrimers [174]. Fourth- and fifth-generation PEGylated PPIs also demonstrated an increase in the entrapment of RIF [175]. Furthermore, targeting of AM was studied using RIF with fifth-generation ethylenediamine (EDA)-PPI dendrimers based on mannosylation to selectively target macrophages. The outcome was that the concentration of the drug in the macrophages exceeded the plasma concentration, making it a promising approach for the treatment of TB [85].

Poorly aqueous or hydrophobic drugs can be encapsulated within the core of PEHAM dendrimers to improve their solubility, which further increases the bioavailability of the drug [176, 177]. Since the cationic groups present in the PEHAM dendrimers are toxic to RBCs, methods such as glycosylation and acetylation are extensively employed to overcome the toxicity [178, 179]. ATDs can also be encapsulated in polymers to increase their half-life and attain enhanced bioavailability and a sustained release profile [180].

#### Polymeric-microparticulate drug delivery systems

The inclusion of RIF and INH into polylactic acid microparticles (MPs) at a 1:1 ratio increased the drug concentration in macrophages, lowering the dosage frequency and toxicity [181]. For the detection and treatment of pulmonary TB, monocyte-derived MPs can be utilized to target AM. To release entrapped pharmaceuticals, sodium alginate, a linear copolymer of α-guluronic acid and α-mannuronic acid, creates a meshwork with divalent cations [182]. Recently, β-1,3/1,6 glucan particles (GPs) produced from yeast have been used to deliver anti-TB medicines to macrophages [183, 184]. Macrophages and other phagocytic cells can recognize the β-1,3-D glucan surface makeup. Particulate glucan is biodegradable and biocompatible, and the United states Food and Drug Administration (FDA) considers it generally recognized as safe (GRAS). In a study, RIF DPI was developed that had the ability to overcome drug resistance as well as reduce the time needed for therapy [185].

INH-administered mannitol microspheres containing iron(III) trimesate metal organic framework (MOF) MIL-100 nanoparticles demonstrated adequate encapsulation efficiency and aerodynamics for pulmonary delivery. In vitro testing in human alveolar adenocarcinoma basal epithelial cells revealed effective internalization, indicating that it is suited for deep lung ATD administration [80]. Fucoidan microparticles loaded with ATDs demonstrated good affinity, aerodynamic features, and no cytotoxicity to lung epithelial cells or THP-1 macrophages [186]. ATDs suppressed 95% of microbial growth and triggered cytokine-mediated macrophage activation even when used as a single formulation [187]. Chitosan polymeric MP loaded with INH and rifabutin showed comparable and increased efficacy against *M. bovis* [188].

Host defense peptide (HDP) microencapsulation with INH demonstrated enhanced and additive antimycobacterial effects, resulting in a lower dosage concentration. Antimycobacterial action was also demonstrated by encapsulated astaxanthin [189, 190]. Following oral dosing, epigallocatechin gallate (EGCG) exhibited a modest therapeutic effect, while microencapsulated EGCG with trehalose sugar (EGCG-t-MS) demonstrated dose-dependent death of TB bacteria in mouse macrophages. In vivo, pulmonary delivery of EGCG for 6 weeks resulted in lower bacterial loads, less inflammation, and fewer granulomas than orally administered EGCG. Combination therapy with EGCG-t-MS with a subtherapeutic dose of regular ATDs demonstrated efficacy comparable to full-dose therapy [191].

The bioavailability of RIF-loaded poly lactic co glycolic acid (PLGA) microsphere powders after intratracheal aerosolization was 92%. Other RIF, INH, pyrazinamide, rifabutin, and linezolid microencapsulated formulations have been studied as prospective NDDSs for prolonged release, infrequent dosage, and adequate bioavailability [192, 193]. Anisimova et al*.* discovered that INH, streptomycin, and RIF encapsulated in poly(butyl cyanoacrylate) (PBCA) and poly(isobutyl cyanoacrylate) (PIBCA) accumulated more intracellularly in monocytes than free drugs [148]. INH, streptomycin, and RIF encapsulated in PBCA and PIBCA accumulated intracellularly, producing a more effective response [131]. Table 3 represents a summary of drug delivery systems for anti-tubercular drug combinations and new approaches.

**Table 3:** Drug delivery systems for anti-tubercular drug combinations and new approaches

Drug/Therapy	Delivery system	Key findings	References
INH and RIF	Liposome	↑ Survival; ↓ Colony-forming-units (CFU) and root specific lung weight	[116]
	Polymeric micelles	Sustained drug release; ↑ activity against resistant and sensitive strains with minimal hemolytic toxicity	[197]
		↑ Drug penetration; diffusion based drug release	[143]
	Graphene oxide coated with chitosan and gum tragacanth	Simultaneous loading of drugs; retaining the efficacy while using graphene oxide	[198]
	3D Printed scaffold	Prolonged drug release; no effect on hepatic and renal functions; consistent maintenance of mic	[199]
		↓ pH-dependent degradation of drugs; ↑ clinical efficiency by using fixed dose combinations	[200]
	Mesoporous silica nanoparticle with polyethyleneimine for RIF and with cyclodextrin-based pH-operated valves	Intracellular release of high concentrations of antitubercular drugs; the pH sensitive valves open only at acidic medium to release the drug	[201]
INH and Rifabutin	Polylactic acid microparticles	Targeting of macrophages, not epithelial cells on inhalation	[181]
	Inhalable fucoidan microparticles	↑ Association frequency; ↓ Cytotoxicity to lung epithelial cells	[186]
INH and Ciprofloxacin	Ligand anchored pH sensitive liposomes	↑ Drug release and macrophage uptake to macrophage; ↑ Drug accumulation in the lung	[119]
INH, RIF and Pyrazinamide	Microemulsion	Dissolution and release studies of drugs showed the release order as INH > Pyrizinamide > RIF	[202]
	Microemulsion	INH and pyrazinamide shows diffusion mechanism while RIF exhibits anomalous release mechanism	[203]
INH, RIF, pyrazinamide and ethambutol	Dendrimer with PEA polymer	↑ Bioavailability; sustained release	[180]
	80% PVA—20% chitosan hydrogel matrix	↑ Drug loading efficiency, extended drug delivery	[204]
		Slow and extended drug release	[205]
	TB-gel	Entrapment of the four major drugs; half of the dose is only required to achieve the same therapeutic action as that of free drug	[18]
INH and pyrazine-2-carbohydrazide	Graphene oxide carbon nanotubes	↓ Cytoxicity; doesnot interact with cellular cycles of Hep-2 cells	[206]
INH with synthetic host defense peptides	Microencapsulation	Additive antimycobacterial activity due to augmentation of membrane penetration by host defense peptide	[189]
INH and fluoxetine	Multiwalled carbon nanotube nanofluid	Additive drug effect with improved antimycobacterial activity	[207]
RIF and Ofloxacin	Niosome	↑ Entrapment efficiency and controlled release till 15 days	[208]
RIF and Levofloxacin	Cyclodextrin	Intracellular release of hydrophobic drugs in macrophages	[161]
RIF and dihydroartemisinin	Chitosan	Overcome *M. tuberculosis*’ resistance to RIF; additive effect	[146]
RIF/INH and Usnic acid	Liposome	Synergistic interaction between RIF and usnic acid	[209]
Pyrazinamide and metronidazole	Thermotropic liquid crystal embedded in cellulose nitrate membrane	The drug penetration is thermos-dependent in nature	[210]
D-cycloserine and Ethionamide	Niosome	↓ MIC; ↑ drug release and entrapment efficiency	[130]
Prednisolone	PEGylated PPI dendrimers	uniform biodistribution of drug in vital organs	[174]
Protein based vaccine	Nanoemulsion	↑ Potency and thermostability due to addition of adjuvants	[211]
N′-Dodecanoylisonicotinohydrazide	liposome-in-Hydrogel system	Thermoresponsive and self-healing properties useful for intra-articular administration for bone TB therapy; rapid drug release into synovial fluid after localized injection, followed by a steady-state drug release	[118]
Photodynamic antimicrobial chemotherapy	Zinc phthalocyanine-liposomes	Inactivation of both sensible and resistant strains of *M. tuberculosis*	[121]
Epigallocatechin gallate with trehalose	Microencapsulation	Dose dependent killing and time dependent killing; ↓ bacterial loads; no granulomas, lesion or inflammation developed	[191]
Eugenol	Diluted solution	Antimycobacterial effect; synergistic effect when combined with other ATDs	[212]
Macozinone	Capsule	Inhibit the enzyme decaprenylphosphoryl-β-d-ribose 2’-epimerase (DprE1) involved in the synthesis of the mycobacterial cell wall	[213]
β-sitosterol	Capsule	Counteract the side effects caused by long-term ATD therapy; Showed improvements in the levels of hemoglobin, neutrophil, creatinine, and urea, with eventual weight gain and higher lymphocyte and eosinophil counts, as compared to the placebo	[214]
Astaxanthin	Microencapsulation	Cost effective natural component with potent antimycobacterial activity	[190]
Calcium phosphate nanocontainers filled with 1,3‐benzothiazin‐4‐one‐043	Microemulsion	Significant antibacterial activity	[215]
1,5-diarylpyrrole and 1,5-diarylpyrazole	Nanoemulsion and niosome	Comparatively potent antimycobacterial activity exhibited by noisome of 1,5-diarylpyrazole than nanoemulsion of 1,5-diarylpyrrole	[128]

Floating drug delivery methods are low-density devices that allow the drug to float on top of the stomach juice, boosting retention time and bioavailability. Quercetin-loaded RIF floating microspheres were developed to treat TB and maintain RIF release in the stomach, and they were found to be stable after six months [194]. RIF stability can be increased by integrating it into sustained-release microporous floating microspheres and gastric-resistant INH sustained-release microspheres. Microporous floating sustained release microspheres were created using emulsification and evaporation techniques, resulting in increased RIF bioavailability [195]. Because one drug can be enteric coated to release in the stomach and the other in the ileum, floating delivery systems are useful for ATD fixed-dose combos. Studies have shown that more RIF is absorbed from the stomach even in the presence of INH [196].

### Inorganic nanoparticles

#### Gold nanoparticles

**Fig. 4:**
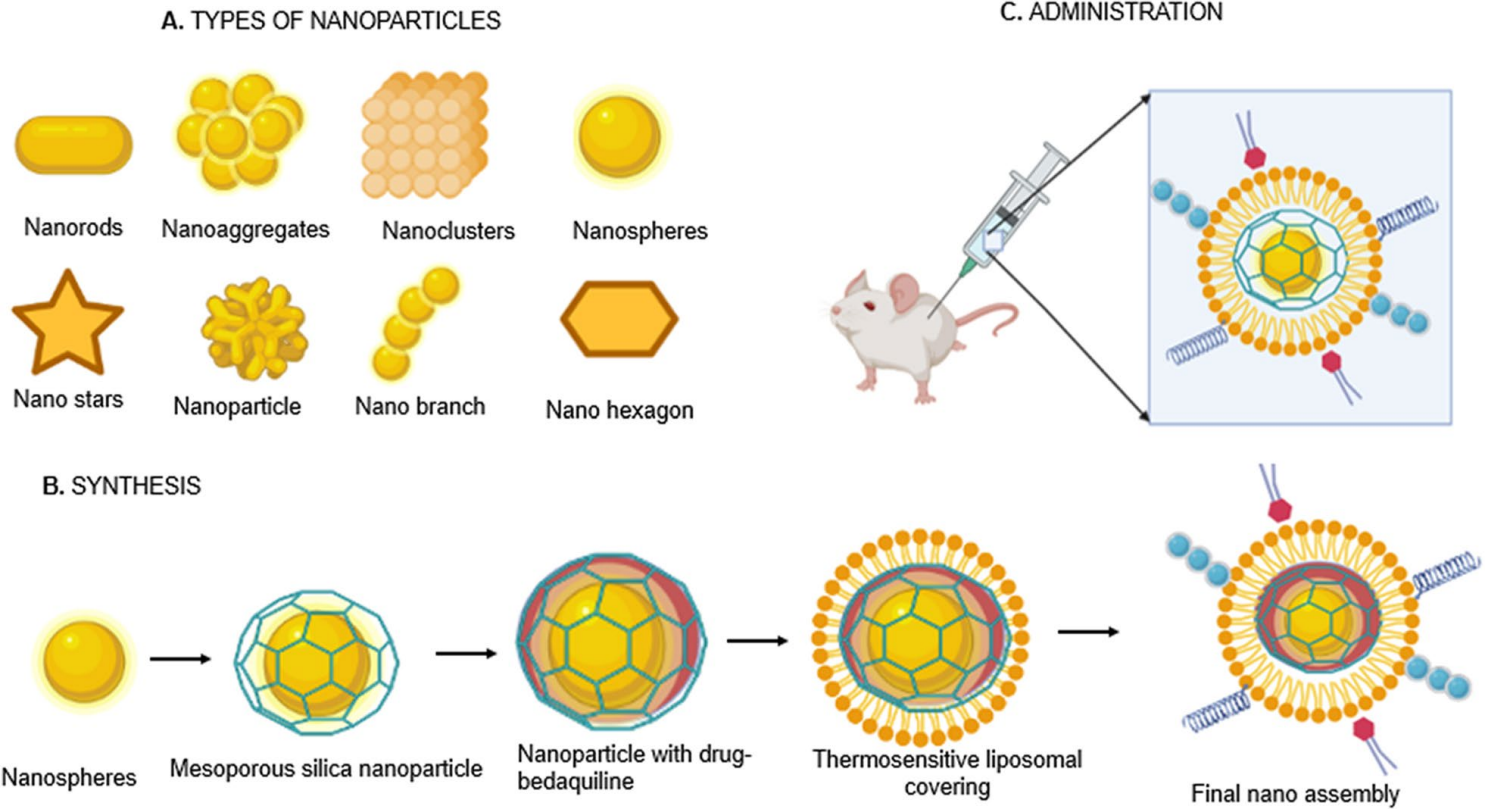
Types of gold nanoparticles and synthesis method. Various types of gold nanoparticles are used for the delivery of ATDs. **A** GNPs are synthesized in various shapes according to the requirements of the delivery system. **B** The synthesis of nanoparticles follows a systematic sequence of steps and can be surface modified to target the nanoparticle to the desired site. **C** The systemic administration of GNPs in preclinical models has been found to enhance the efficiency of drug delivery, thereby improving the action of ATDs

Gold nanoparticles (GNPs) have several applications in targeted drug delivery due to their small size, biocompatibility, and lack of cytotoxicity. Green synthesized GNP with herbs may be beneficial in the treatment of TB. The various types of GNPs and their general synthesis methods are depicted in Fig. 4. GNP exhibits bactericidal activity [216]. With respect to TB, GNP was able to inhibit *M. tuberculosis* with an MIC of 10 µg/ml but was ineffective against RIF-resistant *M. tuberculosis* [217]. Gold nanoparticles synthesized using the bacterium *Zoogloia ramigera* exhibited good antibacterial activity and can be utilized for TB treatment. The antibacterial properties were studied using MIC and minimum bactericidal concentration methods [218]. Mesoporous silica nanoparticles (MSNs) containing gold nanoparticles (MSNs@GNP) can inhibit *M. tuberculosis* growth and produce a synergistic effect against *M. tuberculosis*, making it safe for TB treatment [219]. Another important application of GNP is in TB diagnosis. A ferromagnetic GNP-based immune detection system was developed for the detection of *M. tuberculosis* and to differentiate *M. bovis* [220]. GNPs loaded with quadruplex DNA motifs can aid in the diagnosis of *M. tuberculosis* in sputum [15, 221]. Magnetic beads and GNP-based immuno-PCR assays were developed to detect *M. tuberculosis* antigen [222].

#### Silica nanoparticles

Silica nanoparticles have the ability to be taken up by macrophages and produce immunological benefits [223]. Polyethyleneimine (PEI)-coated MSNs loaded with RIF exhibited effective targeted intracellular delivery with decreased cytotoxicity [224, 225]. MSNs loaded with first-line ATDs can kill *M. tuberculosis*-infected macrophages [201, 226]. MSNs containing NZX (mycobacterial peptide) can effectively treat TB by killing MDR strains of *M. tuberculosis* [227]. Tenland et al*.* found that MSNs can increase antibacterial activity against *M. bovis* and *M. tuberculosis* H37Rv in vitro and in vivo [227]. NapFab, an antimicrobial peptide isolated from bronchoalveolar lavage, showed excellent antimycobacterial activity when introduced into dendritic MSNs. MSNs can be used as carriers for the delivery of silver nanoparticles to target sites because they have very high bactericidal potency. A 2D hexagonal MSN containing silver bromide also showed good antimycobacterial activity [228].

*M. tuberculosis* produces extracellular vesicles that can cause immunomodulatory responses. Nanodrug delivery systems such as MSNs can mimic endogenous vesicles and act as carriers of the vesicle-associated proteins Ag85B, LprG, and LprA. They have been studied for the development of vaccines against TB [229]. Acetophenone helps MSNs deliver clofazimine to the target site [230]. Oral drug delivery of antitubercular nitroimidazopyrazinone analogs-pretomanid and MCC7433 from the bicyclic nitroimidazole class can be improved by MSNs. The MCM-41 type of SNP was used as a carrier for the transport of poorly water-soluble bicyclic nitroimidazole compounds [231]. MSNs are a promising multifunctional drug delivery system due to their high drug loading capacity and stability [232].

### Carbon nanotubes

Carbon nanomaterials have gained popularity due to their unique features, which include physiochemical, thermal, optical, and electrical properties. Carbon nanotubes are the most well-known structures with a continuous cylinder formed of graphene [233]. Zomorodbakhsh et al*.* 2020 linked INH with multiwall carbon nanotubes (MWCNTs), which demonstrated greater lethality against *M. tuberculosis* even at considerably lower concentrations than the free drug [79]. Chen et al*.* developed chitosan nanotubes based on INH nanoparticles to increase medication release time, accelerate TB ulcer healing, and minimize inflammation and cytotoxicity [78]. Tudose et al*.,* Moradi et al*. and* Pi et al*.* used a graphene oxide carrier system with various surface modifications for the delivery of ATDs and were found to have superior control over drug release [97, 198, 206].

**Fig. 5:**
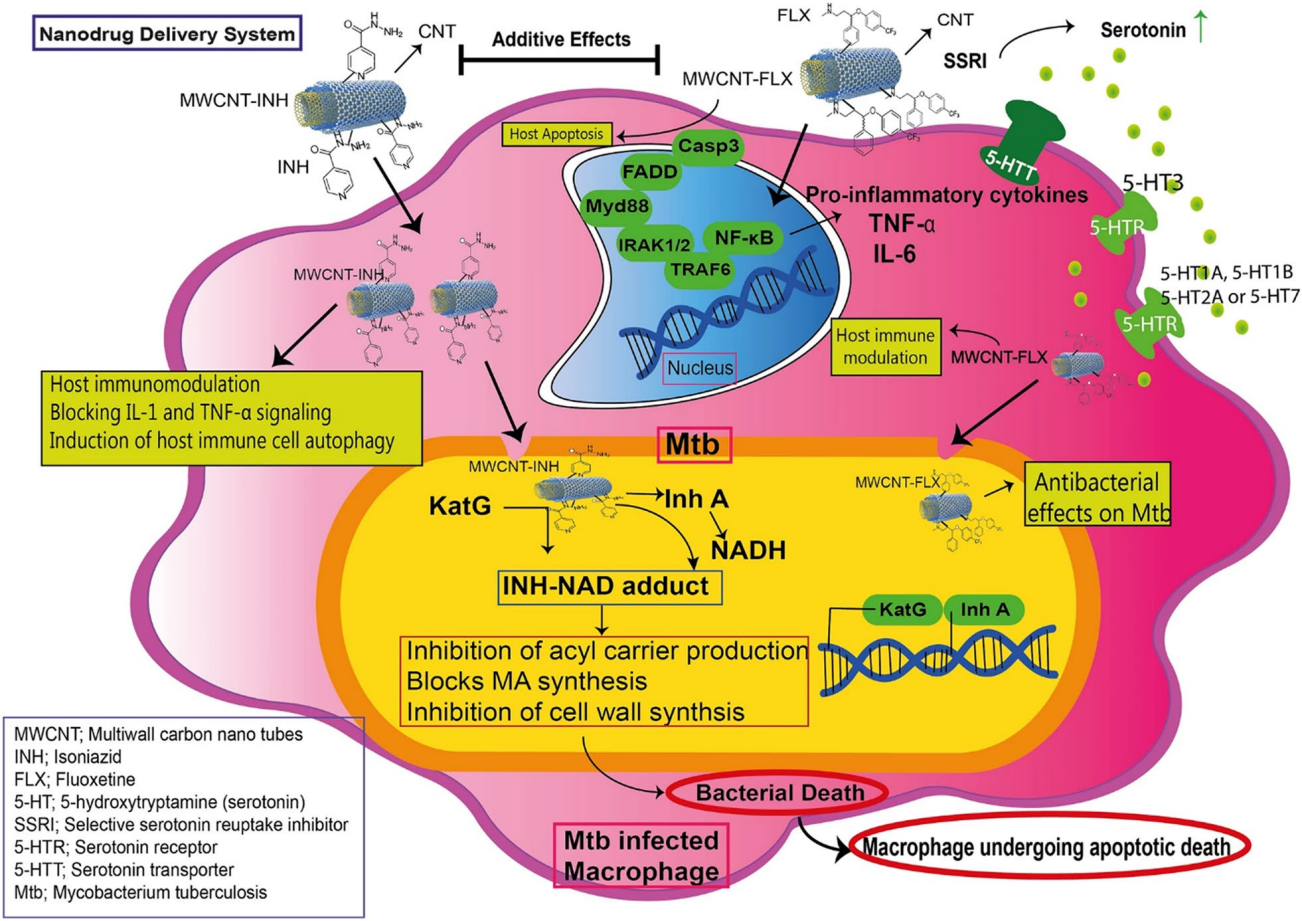
Chemical structures of the MWCNTs conjugated with therapeutic molecules and aspects of the release of cargos. Reprinted from [207] CC BY license

More et al*.* developed a graphene oxide-based air-dried hydrogel containing para-amino salicylic acid for targeting MDR TB, whereas Vatanparast et al*.* revealed that AlN- and AlP-doped graphene quantum dots (GQDs) can be utilized to transport INH [7, 77, 114]. INH- and fluoxetine-conjugated MWCNTs increased the antimycobacterial activity and were capable of regulating the expression of the INH resistance genes inhA and katG, as shown in the schematic representation in Fig. 5 [207]. Carbon nanomaterials have been used to create electrochemical biosensors for *M. tuberculosis* detection, such as an amperometric DNA biosensor and a microfluidic multiplexed platform based on carbon nanotubes [234].

### Emulsion-based drug delivery systems

#### Microemulsions

Microemulsions can be utilized to deliver targeted drugs, control release, and improve ATD bioavailability. Mehta et al. [202] formulated a highly stable Tween 80 microemulsion using RIF and INH [235]. Tween-based microemulsions of ATDs such RIF, INH, and pyrazinamide were found to be less hazardous and irritating, with INH releasing faster in the continuous phase and RIF releasing faster in the droplet phase [202]. The encapsulation efficiency, shape, antimycobacterial activity, particle size, and zeta potential have all been improved using modified microemulsion procedures [100, 236]. These medication delivery technologies can significantly reduce dose frequency while increasing bioavailability. Kaur et al*.* developed a Brig 96 microemulsion to work in combination with lipophilic and hydrophilic drugs [203]. Microemulsions of INH, pyrazinamide, and RIF exhibited good antibacterial characteristics [237].

Using the microemulsion process, calcium phosphate nanocontainers can be loaded with 1,3-benzothiazine-4-one, a new antimycobacterial drug that results in an increase in local drug concentration at the site of mycobacterial infection. Eugenol, an active chemical ingredient, was also combined with Tween 20 as a surfactant to improve therapeutic efficacy against *M. tuberculosis* [238]. According to Talegaonkar et al*.*, microemulsions enhanced drug solubility and absorption, making RIF more effective and less toxic [239]. Microemulsions were also discovered to be promising for the regulated administration of ATDs as well as the destruction of drug-resistant strains of *M. tuberculosis* [240].

#### Nanoemulsion

*M. tuberculosis* can affect the eyes and cause an ocular infection resulting in permanent vision loss [98]. In this case, ATDs have to overcome the obstacle of the blood‒retinal barrier for effective drug movement, decreasing bioavailability. A solution could be the formulation of drugs into nanoemulsions by using excipients such as chitosan and polymyxin B [241]. For instance, a cationic RIF nanoemulsion was produced by high-pressure homogenization in a way that does not affect the therapeutic efficiency of the drug while enhancing bioavailability and other pharmacokinetic parameters [98]. Nanoemulsions were also used to develop a thermostable adjuvanted vaccine against TB by the "design of experiment (DoE)” approach [211].

### Hydrogels

Hydrogel-forming microneedle arrays were designed for the transdermal delivery of ATDs, enabling the administration of high doses of ATDs [242]. Transdermal injection of hydrogel-based medicines has been proven to improve antibiotic activity against *M. tuberculosis* infection. For in vitro permeation, three distinct drug reservoirs were developed and combined with hydrogel-forming microneedle arrays. When the microneedle arrays were paired with polyethylene glycol tablets, immediately compressed tablets, and lyophilized tablets, the maximum penetration of RIF, ethambutol, INH, and pyrazinamide was attained [81].

More et al*.* formulated a graphene-based hydrogel that contained PAS and had good biocompatibility and antimycobacterial capabilities [7]. Wan et al*.* synthesized a variety of cationic peptide amphiphiles capable of self-assembling hydrogels [243]. A graphene oxide air-dried hydrogel designed to target *M. tuberculosis* also showed excellent antibacterial activity [7]. Polyvinyl alcohol (PVA) is nontoxic and has a high effectiveness for the encapsulation of hydrophilic pharmaceuticals, and a PVA-chitosan-tripolyphosphate hydrogel was developed for the extended release of ATDs. In a phosphate buffer solution (pH 7.4), a formulation of 80% PVA—20% chitosan hydrogel matrix demonstrated the maximum rate of drug release in a short period of time, with varied release patterns for RIF, INH, ethambutol, and pyrazinamide [204, 205].

Hydrogel interconnecting micelles made with guar gum/chitosan/polycaprolactone can serve as effective carriers for poorly water-soluble medicines such as RIF [142]. TB-Gel is an injectable and nonimmunogenic amphiphilic-based drug delivery technology with a low molecular weight. In an experimental mouse model, it was found to be more effective than oral delivery of a combination of four medicines in lowering mycobacterial infection [18]. Because of their swelling behavior, wide size range, and biocompatibility, hydrogels are effective in resolving these problems [244].

### 3D-printed formulations

**Fig. 6:**
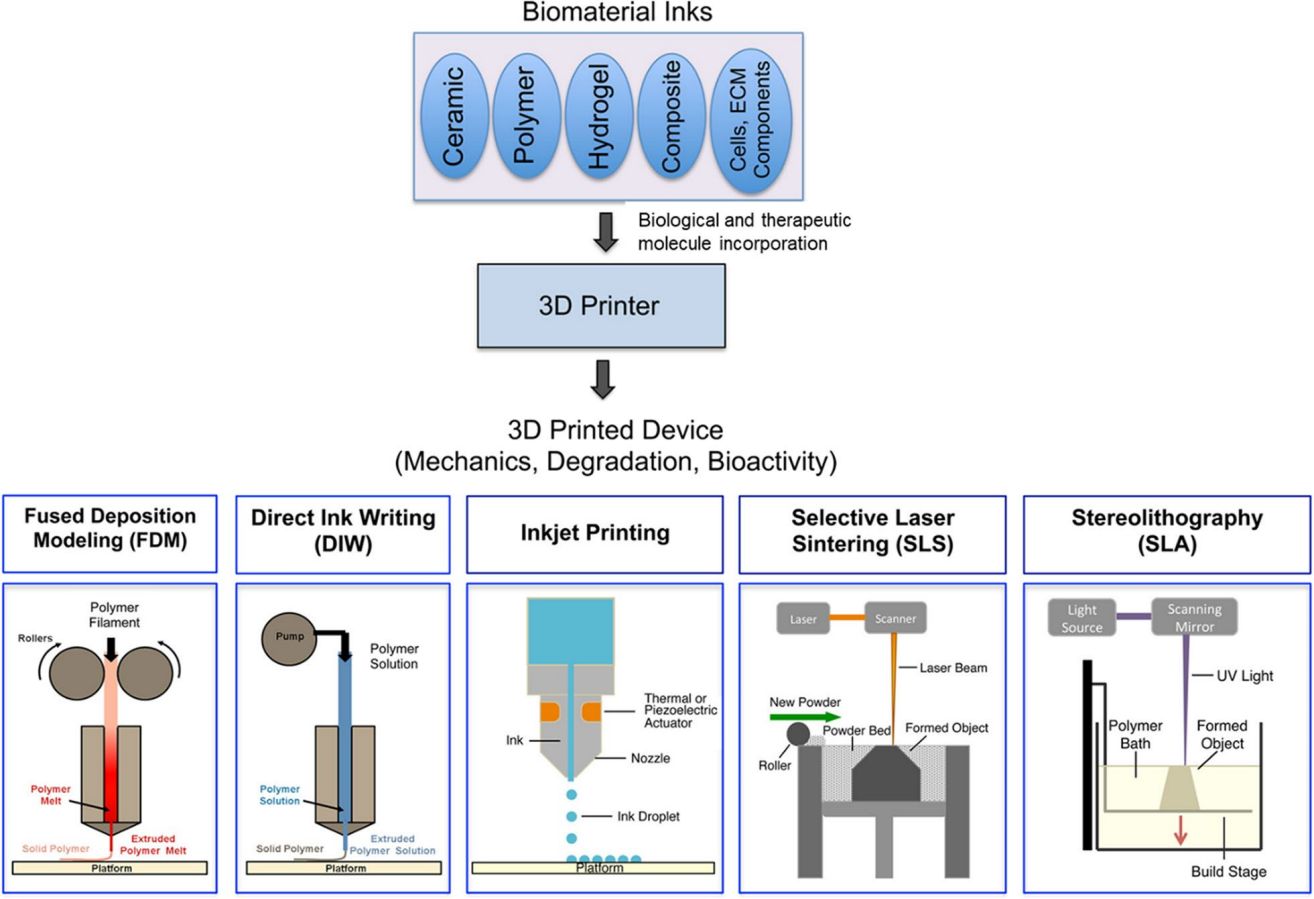
Possibilities with 3D printing techniques: Various biopolymers and ceramics can be incorporated with therapeutics by utilizing various 3D printing techniques. Modified with permission from [245] © American Chemical Society

3D printing techniques could truly advance the application of modern drug delivery systems in TB treatment by precision in the fabrication of scaffolds with well-controlled inner structures and pore morphologies, as depicted in Fig. 6 [245]. The prospects could be enhanced by the use of biocompatible and biodegradable polymers, such as polycaprolactone, as the binder for 3D printing [246].

A 3D-printed scaffold containing drugs such as INH and RIF can minimize the occurrence of drug resistance in osteoarticular TB [199]. In osteoarticular TB debridement procedures, drugs are placed into mesoporous and bioactive ceramics that bond with poly(3hydroxybutyrate-co-3-hydroxyhexonate) (PHBHHx) [246].

A bioengineered delivery system, such as 3D-printed tablets with discrete compartmentalization for RIF and INH, has the advantage of reducing drug degradation, allowing for successful combination treatment [247]. Bilayered tablets with two different ATDs, INH and RIF, can be designed and manufactured using a 3D-printed scaffold. The pH-sensitive polymer in which these medicines are contained determines the site of release. These methods can reduce pH-dependent deterioration and improve therapeutic efficiency [200].

Quercetin, a flavonoid, has been found to limit the growth of *M. tuberculosis* H37Rv. To combat the devastating effects of pulmonary TB, 3D printing technology was employed to create medicinal skin patches. Pharmacokinetic studies in rats revealed the viability of creating 3D-printed medicinal skin patches that may deliver plasma levels for up to 18 days following a single application [248, 249].

## Advanced therapeutic strategies

### Targeted therapy

**Fig. 7:**
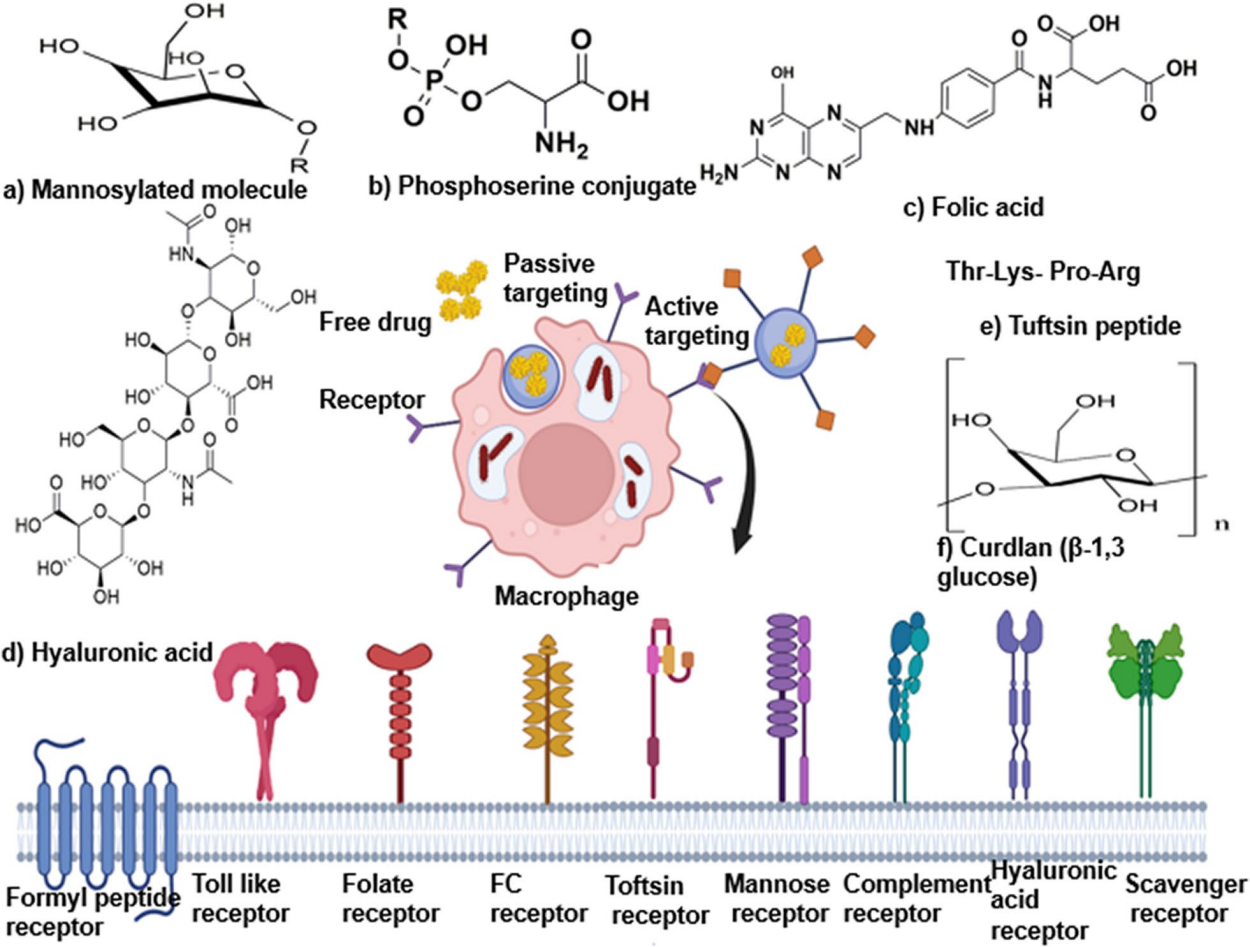
Targeting infected alveolar macrophages (AM). In active targeting, ligands are incorporated into the drug carrier, which interacts with specific receptors on AM, leading to ligand‒receptor-mediated phagocytosis. In passive targeting, the surface of the carrier lacks a host-specific ligand. The macrophage surface receptors that can be utilized for active targeting include the formyl peptide receptor, Toll-like receptor, folate receptor, Fc (fragment, crystallizable) receptor, tuftsin receptor, mannose receptor (CD206), complement receptor, hyaluronic acid receptor (CD44), scavenger receptor, fucosyl receptor, Dectin-1 receptor and lectin-like receptors. Common ligands used to target macrophages include **a** Mannosylated molecule, **b** Phosphoserine conjugate, **c** Folic acid, **d **Hyaluronic acid, **e** Tuftsin peptide, **f **Curdlan (β-1,3 glucose). Concept adopted from [30]

Among polymers, biocompatible PLGA has found profound use in the fabrication of controlled ATD delivery systems because of the ease in achieving the desired dose and release kinetics by modification of the lactide to glycolide ratio, molecular weight, and drug concentration [250–252]. With regard to tissue-resident macrophage targeting for the delivery of ATDs, the surface of the drug carrier is functionalized with ligands, including mannosylated molecule, β-glucan, curdlan (β-1,3 glucose), folic acid, hyaluronic acid, tuftsin peptide, and phosphoserine conjugate, that can be recognized by corresponding receptors on macrophages, such as mannosyl receptor (CD206), dectin-1 receptor, folate receptor, tuftsin receptor, hyaluronic acid receptor, fucosyl and scavenger receptor, Fc receptor, transferrin receptor, formyl peptide receptor (FPR), and other lectin-like receptors [30, 253, 254] represented in Fig. 7. Among them, drug carriers made of modified mannose are the most common and can be added to liposomes, SLN, and polymer micelles [255–257] Studies have shown that cubosomal lipid nanocarriers exhibit higher drug delivery efficiency as well as bioavailability than conventional formulations. They are not only effective against free bacilli but also have the ability to deliver drugs to intracellular bacilli [258]. Thus, the identification of better sets of ligands with higher binding affinity for macrophage-based receptors might result in enhanced targeting efficiency. A promising approach could be heteromultivalent targeting, in which different types of ligands bind to different macrophage receptors simultaneously [259]. With respect to targeting and drug accumulation at sites of infection, future studies need to focus on the translation of preclinical data into humans in relation to the severity of infection, the fraction of drug at off-target sites, and the drug targeting index [14, 260].

Pulmonary TB is the most common form of TB, and inhalable carriers of ATDs have been a major focus of research and were found to significantly increase targeting in the lungs, reducing undesirable toxic side effects and enabling delivery to AM [9, 14]. Liposomes [261], microparticles [186, 187, 262], microencapsulation [80], liquid crystals [263], hydrogels [244], polymeric micelles [96] and even hybrid systems [264] have been promising for inhalational TB therapy. The formulations need to be optimized to penetrate mucous layers and biofilms and overcome sequestration, rapid deactivation by enzymes, and elimination by coughing [265, 266]. In addition to passing through the acidic gastric environment, pulmonary administration could be particularly beneficial for controlled release within AM. However, for translatable pulmonary administration of ATDs, challenges need to be resolved, including the use of better and safer excipients, drug encapsulation efficiency, process and production scalability, and developing formulations with optimum size and morphology for deep lung deposition [124, 267–269]. If sufficient drugs can be delivered through the inhaled route, it would be of immense benefit to TB patients considering the acceptance of portable, cost-effective and easy-to-operate inhalers [23].

Unlike the conventional administration of drugs, nanomaterial-based systems offer significant benefits, such as ease of administration, minimal side effects, and addressing the pharmacodynamics and pharmacokinetic limitations of many potential drug molecules [270]. Recently, transforming growth factor (TGF)-β1-specific siRNA nanoliposomes loaded with INH, RIF, and pyrazinamide have demonstrated the potential for improving spinal TB chemotherapy [271]. With the adoption of a phage-based delivery system for endogenous type III-A CRISPR‒Cas antimicrobials against *M. tuberculosis*, nanoenabled CRISPR‒Cas-powered strategies might also be developed for the treatment of TB [272–274]. Thus, nanotechnology holds immense potential in developing novel and targeted delivery systems for new therapies as well as existing drugs. This could be of interest, particularly in the development of inhaled or orally delivered nanocarriers for extended release of ATDs, which in turn can reduce the required dosing frequency to improve patient adherence. Drug depot systems can be of remarkable benefit, particularly for the treatment of TB in children, if balance can be achieved between the ease and safety of administration [275–277].

### Long-acting therapeutics

Long-acting therapeutics can be formulated using prodrugs that have low aqueous solubility, inhibit rapid dissolution and drug release, and have a reasonably long half-life, enabling slow elimination from the body and high potency, allowing low drug doses to be injected [278–280]. In this regard, drugs such as bedaquiline, which has a longer half-life (24 h), higher lipophilicity (logP 7.3), and lower MIC for *M. tuberculosis* (0.03 μg/ml), were found to be suitable for use in a long-acting injectable (LAI) formulation [278, 281, 282]. Remarkably, one intramuscular injection of long-acting bedaquiline at 160 mg/kg demonstrated significant antitubercular activity for 12 weeks in p mouse models. Moreover, physiologically based pharmacokinetic modeling identified delamanid and rifapentine as potential LAI candidates suitable for monthly intramuscular administration at doses of 1500 mg and 250 mg, respectively [282]. A promising development is the one-time large-dose controlled release delivery system resident in the gastrointestinal tract. It offers numerous advantages over currently available injectable depot formulations, including ease of administration, lack of immunologic reactions, and the ability to accommodate multigram-level dosing in line with current TB treatment regimens [283].

In another approach, a thermoresponsive matrix containing an extended-release polymer was used to encapsulate drug molecules to improve the duration of action. Significant efficacy was achieved using sustained release intramuscular injection loaded with tin protoporphyrin (SnPPIX), a heme oxygenase-1 inhibitor, in murine models of pulmonary TB [284].

Furthermore, notable technological advancements for TB include developments in oral and subcutaneous systems [285]. Solid nanoparticles (SNPs) are widely studied for the oral delivery of antimicrobials, including SLNs, polymeric nanoparticles, MSNs, and hybrid nanoparticles [286]. SLN has been tested in rodents with the goal of improving the bioavailability of RIF and the combination of INH, RIF, and pyrazinamide [14, 287]. MSNs were able to enhance the activity of orally delivered poorly soluble antibacterial agents against TB, such as pretomanid and MCC7433, a novel nitroimidazopyrazinone analog [231]. SLN was also proposed as a suitable drug delivery platform with short-term sustained release upon intramuscular and subcutaneous administration [288].

### Extrapulmonary TB therapy

ATD delivery for TB bone defects has been another area of focus in TB research, as bone TB has the highest incidence among extrapulmonary TB, accounting for approximately 35–50%. Even though surgery is available, the remaining *M. tuberculosis* around the trauma can multiply, leading to TB ulcer and sinus formation and even causing bone TB recurrence and bacterial infection. This, along with poor local blood supply that causes difficult access to ATDs, complicates the scenario [289]. A probable solution could be loading ATDs into scaffolds through various drug-loading techniques to improve the efficiency of anti-TB treatment [290]. As carbon nanotubes have strong penetrability across physiological barriers to enter tissues, chitosan/carbon nanotube nanoparticles were constructed to achieve slow release of INH. It was found to significantly promote the healing of TB ulcers and could be developed as a new treatment for secondary wounds of bone TB [78].

Another approach that has shown promising potential for osteoarticular TB therapy is biocompatible mesoporous bioactive glass/metal–organic framework (MBG/MOF) scaffolds fabricated by a 3D printing technique using polycaprolactone [246]. MBG is considered a promising material owing to its bone repair potential in relation to its high surface area and better bioactivity, along with its superior drug loading and release ability [291–293]. The biomedical applications of MOFs are related to their tunable porosity, biocompatibility and biodegradability, making them an attractive drug delivery system with a modifiable degradation rate for controlled drug delivery [294, 295].

In comparison, the cutaneous administration of ATDs is poorly explored. Skin can be considered a good route for the treatment of cutaneous TB, usually caused by atypical mycobacterium species, namely, *M. leprae*, *M. hemophilum*, and *M. ulcerans.* TB represents only 1–1.5% of extrapulmonary cases, affecting mainly the face, torso, and neck areas [296]. However, the incidence of extrapulmonary TB is increasing in the context of MDR-TB. Recently, van Staden et al*.* proposed the utility of self-double-emulsifying drug delivery systems (SDEDDS) containing clofazimine for topical delivery in the treatment of cutaneous TB [297]. The benefit of SDEDDS for dermal administration of clofazimine is that, with lower drug concentrations, it could provide consistent drug delivery profiles that will be cytotoxic toward *M. tuberculosis,* which can help to suppress drug resistance [298]. As a topical delivery, it might be able to reduce the unpleasant discoloration associated with oral administration of clofazimine [299]. The improved drug loading capacity of SDEDDS may be further utilized to treat active TB or resistant TB infections by either including higher concentrations of clofazimine or incorporating fixed-dose drug combinations with other drugs known to act synergistically [300].

The possibility of INH delivery by the skin route has also been evaluated, as this route could avoid the hepatic first-pass effect, thereby reducing hepatotoxicity that leads to poor patient compliance [301]. The selection of excipients is based on the intended use. For example, limonene was found to be the better excipient for transdermal formulations based on the enhancement of INH absorption, while transcutol and menthol were found to be more appropriate for topical systems. Inclusion of transcutol led to increased skin accumulation of the drug, termed the "intracutaneous depot", created by swelling of stratum corneum intercellular lipids that retained the drugs, along with a simultaneous decrease in transdermal permeation. Interestingly, the incorporation of limonene resulted in transdermal absorption of INH that was sufficient to ensure a systemic therapeutic effect [301]. Moreover, to achieve transdermal drug delivery, cutting-edge anti-TB drug delivery systems are being explored, such as 3D printed quercetin-coupled polyvinylpyrrolidone (PVP) skin patches for the treatment of destructive pulmonary TB [249].

Another avenue is ophthalmic drug delivery for the treatment of ocular TB, in which eyes and orbital tissues are affected, leading to ocular morbidity and visual loss [98, 302]. Even though it is a comparatively rare extrapulmonary manifestation, ocular TB may be the first presentation of TB in initially asymptomatic patients, especially since 92% of patients with ocular TB present without evidence of concomitant pulmonary TB [303–305]. A RIF-loaded cationic nanoemulsion with specific surface modification employing chitosan and polymyxin B was found to be promising to overcome the hurdle of the blood‒retinal barrier of the eye that hinders the availability of ATD delivered by the systemic route [30, 98].

### Phototherapy

An emerging technology with promising application in TB therapy is combining ATDs with other treatment modalities, such as photodynamic and photothermal therapies [306, 307]. For instance, a targeted antibiotic-delivering nanoassembly was shown to exert chemo-photothermal therapy [307]. The core of the nanoassembly was composed of near-infrared (NIR) active gold nanorods (GNRs) coated with MSNs, which served as the carrier for bedaquiline. The assembly was wrapped within a thermosensitive liposome (TSL) conjugated to the mycobacteria-targeting peptide NZX, which mediated adhesion of the final nanoassembly on the mycobacterial surface and had intrinsic antibacterial activity. Upon NIR exposure, TSL undergoes a phase transition, becoming permeable due to the heat generated from the GNRs, releasing encapsulated bedaquiline. Hyperthermia also plays a role in increasing bacterial cell membrane permeability, causing leakage of bacterial cell contents and subsequent bacterial cell death. The final nanoassembly demonstrated remarkable antibacterial activity against *M. smegmatis*, which was 20-fold more efficacious than the free drug equivalent. Moreover, it successfully inhibited the growth of intracellular mycobacteria residing in lung cells, underlying its potential to treat latent pulmonary TB. The engineered nanoassembly was able to (1) control remote trigger release of encapsulated ATD upon exposure to NIR laser by melting of TSL, (2) increase internalization into infected host cells through TSL coating and offer targeted ATD delivery to the bacterial cell surface by NZX targeting peptide, thereby reducing off-target toxicity, and (3) demonstrate synergistic antibacterial activity due to encapsulated ATD and photothermal activity [307]. Furthermore, combined chemo-photothermal therapy based on an enzyme-responsive nanosystem could be a promising approach to combat drug-resistant bacteria[308]. Even photodynamic therapy could be a new option for the treatment of MDR- and XDR-TB, as it was able to inactivate *M. tuberculosis* clinical strains regardless of the drug resistance levels of the bacilli [8].

### Immunotherapy

The immune system significantly impacts TB recognition, occurrence, development, and outcome. The disease's progression depends on genetics and environmental factors. In the initial stages, innate immune clearance is involved, while macrophages, neutrophils, dendritic cells, T cells, and NK cells form the first line of defense [309]. The interaction between host immunity and mycobacterial invasiveness affects the immune system response. If the invasiveness of bacilli is weak, macrophages eliminate it, generating trained immunity. If mycobacterial invasiveness is balanced with host immunity, bacilli may replicate, spread, and become active TB. TB-specific immunotherapy is needed to regulate the immune system's anti-TB response. Cytokines such as IL-2, IL-24, and IL-32 can be therapeutic targets against TB [310–314]. IL-2, a Th1 immune response cytokine, induces differential gene expression in peripheral blood mononuclear cells (PBMCs) stimulated by TB. Administration of rhuIL-2 immunoadjuvant enhances CD4 + T-cell proliferation and NK cell proliferation, improving the sputum bacterium-negative rate in MDR-TB patients. A multicenter clinical trial on rhuIL-2 as an adjuvant therapy for MDR-TB is being conducted in China (ClinicalTrials.gov Identifier: NCT03069534). IL-24, a novel tumor suppressor, inhibits IL-24 expression in human PBMCs, increasing susceptibility to TB [314–317]. NK cells, T cells, and macrophages play a crucial role in combating TB. IL-24 activates CD8 + T cells, producing IFN-γ and IL-32, which induce inflammatory cytokines such as IL-1, IL-6, IL-8, and TNF-α. Heat-killed TB stimulates PBMCs to produce IL-32, enhancing clearance by monocyte macrophages. Anti-TB antibodies also have protective effects on anti-TB immunity [318–321]. Antimicrobial peptides, small molecule peptides, can enter cells through the skin and placenta exhibiting bactericidal and immunotherapeutic effects on TB [322].

Antigens can alert immune cells and precipitate an immunological reaction [323]. If these antigens can be engineered in such a way that they target proteins of *M. tuberculosis*, such as ESAT-6, CFP-10, and TB 7.7, these antigens can be used effectively against TB bacilli [324]. Accordingly, amino acid polymers that self-assembled to form a hollow core-shaped nanobead were administered to TB patients and produced different cytokines, including IFN-γ, INF-α, IL-2, CCL3, and CCL11 [325]. The biopolyester and polyhydroxybutyrate beads were biocompatible, thereby minimizing adverse reactions. The evaluation of the engineered antigen was performed by interferon release assay [326]. This delivery system could not only be used for ATD delivery but also for TB diagnosis, especially in patients showing tuberculin skin test negative (TSTn) results, as it contains short overlapping synthetic peptides such as in the QuantiFERON-TB Gold in Tube test (QFT-GIT) [327].

## Conclusion and future perspectives

In conclusion, advanced drug delivery approaches have the potential to revolutionize TB therapy by addressing the challenges associated with traditional treatment methods. By developing inexpensive and easy-to-administer delivery systems that offer extended drug release, dosing frequency could be reduced, thereby improving patient adherence. Direct targeting by selectivity toward both AM and tubercle bacilli using suitably designed drug carriers and specific ligands may counteract the ability of intracellular pathogens to evade antibiotic treatments. With better penetration of ATDs into lung cavities and necrotic lesions, the success rate of TB therapy could be increased. In recent years, there has been a rise in TB cases, particularly resistant forms, across the globe. Various strategies for combating TB have been established at different levels, including the WHO’s End-TB Strategy and the UN’s Sustainable Development Goals (SDGs) [328]. The ‘3P Project’ aims to unite researchers to develop a treatment strategy that lasts for at most one month for all forms of TB infections [329]. Remarkably, the Medicines Patent Pool (MPP) has facilitated the clinical development of promising investigational treatments for TB, such as sutezolid, a linezolid analog [330]. In addition to the prospect of new drugs for TB, a favorable approach has been to improve the aspects of drug delivery through technologies that can offer the flexibility to adopt better routes of administration, multiple drug encapsulation, sustained drug release, targeted drug delivery, enhanced permeability and retention along with a lower incidence of side effects [331]**.** This indeed has the potential to overcome patient nonadherence to long and frequent dosing regimens [275]. The challenges that need to be addressed with some of the current ATDs are their poor solubility, instability in gastric acid, and inability to penetrate AM, where the bacilli reside [14, 332].

Recently, a significant breakthrough has been made in the treatment of TB through the use of siRNA-loaded nanoparticles, which effectively silence genes specific to *M*. *tuberculosis*. Additionally, a lyophilized formulation of the emulsion-adjuvanted subunit ID93 with GLA-SE, a recombinant subunit antigen combined with a squalene emulsion containing glucopyranosyl lipid A (GLA), has shown promise in a phase 1 clinical trial (Clinical trials.gov identifier: NCT03722472). This thermostable vaccine formulation demonstrated safety and immunogenicity in healthy adults. Similar technology-based formulations have also undergone clinical trials (Clinical trials.gov identifiers: NCT01599897, NCT01927159, NCT02465216, NCT02508376, and NCT03722472), and updates on these trials and development status can be accessed through The Working Group on New TB Vaccines (WGNV) database (https://newtbvaccines.org/tb-vaccine-pipeline/).

Another formulation, a liposome suspension known as RUTI®, containing a mixture of antigens, is actively recruiting patients for phase 2 clinical trials (Clinical trials.gov identifier: NCT04919239). These advancements highlight the potential of advanced drug delivery strategies in addressing the challenges of TB treatment. Despite the promising research activity, progress in clinical trials has been relatively slow.

To further advance the field, it is imperative to focus on addressing research gaps related to drug delivery systems for TB management. These gaps include the need for targeted delivery to specific cells and tissues, enhancing drug bioavailability, optimizing drug release kinetics from delivery systems, ensuring biocompatibility and biodegradability, addressing immunogenicity concerns, enabling personalized medicine approaches, exploring combination therapy benefits, considering cost-effectiveness, navigating regulatory approval processes, promoting successful clinical translations and validations, and fostering interdisciplinary collaboration.

In conclusion, while recent developments in TB treatment using advanced drug delivery strategies are encouraging, continued efforts are required to bridge the gap between research advancements and clinical application. By focusing on the aspects, the field of TB drug delivery can overcome challenges and contribute to more effective and accessible treatment options for patients worldwide.

## Data Availability

Not applicable.

## Abbreviations

AM: Alveolar macrophages
ATDs: Anti-tubercular drugs
CCL11: Chemokine (C-C motif) ligand 11
CCL3: Chemokine (C-C motif) ligand 3
CDs: Cyclodextrins
CFU: Colony-forming units
CRISPR: Clustered regularly interspaced short palindromic repeats
CS-g-PCL: Chitosan-graft-poly-ε-caprolactone
DNA: Deoxyribose nucleic acid
DoE: Design of experiment
DOTAP: 1,2-dioleyl-3-trimethyl-ammonium-propane
DOTAP: N-(1-(2,3-Dioleoyloxy)propyl)-N,N,Ntrimethylammonium methyl-sulfate
DOTS: Directly observed treatment short-course
DprE1: Decaprenylphosphoryl-β-d-ribose 2’-epimerase
DPI: Dry powder inhaler
EGCG: Epigallocatechin gallate
EGCG-t-MS: Microencapsulated EGCG with trehalose sugar
ELISA: Enzyme-linked immunosorbent assay
FDA: Food and Drug Administration
FPR: Formyl peptide receptor
GLA: Glucopyranosyl lipid A
GNPs: Gold nanoparticles
GNR: Gold nanorods
GPs: Glucan particles
GRAS: Generally Recognized As Safe
GQDs: Graphene quantum dots
HDP: Host defence peptide
HP- β -CD: Hydroxypropyl- β -CD
IFN-γ: Interferon gamma
IL-2: Interleukin 2
I.M: Intramuscular
INH: Isoniazid
INVITE: Inulin functionalized with vitamin E
INVITESA: Inulin functionalized with a vitamin E succinylated derivative
I.V: Intravenous
LAI: Long-acting injectables
MBG: Mesoporous bioactive glass
MDR: Multidrug resistant
MIC: Minimum inhibitory concentration
MO: Monoolein
MOF: Metal organic framework
MP: Microparticles
MPP: Medicines Patent Pool
MSNs: Mesoporous silica nanoparticles
MWCNTs: Multiwall carbon nanotubes
NPs: Nanoparticles
NDDS: Novel drug delivery systems
NIR: Near infrared
NSCLC: Non-small cell lung cancer
PAMAM: Poly(amidoamine)
PBCA: Poly(butyl cyanoacrylate)
PBMCs: Peripheral blood mononuclear cells
PCL: Poly-caprolactone
PEA: Poly(ester amine)
PEG: Polyethylene glycol
PEHAM: Poly(ether hydroxylamine)
PEI: Polyethyleneimine
PEO-PPO: Polyethylene oxide- polypropylene oxide
PHBHHx: Poly(3hydroxybutyrate-co-3-hydroxyhexonate)
PIBCA: Poly(isobutyl cyanoacrylate)
PLGA: Polylactic co glycolic acid
PPI: Poly (propylene imine)
PVA: Polyvinyl alcohol
PVP: Polyvinylpyrrolidone
QFT-GIT: QuantiFERON-TB Gold in Tube test
RIF: Rifampicin
SDEDDS: Self-double-emulsifying drug delivery systems
SDGs: Sustainable development goals
si RNA: Small interfering RNA
SLNs: Solid lipid nanoparticles
SNPs: Solid nanoparticles
TB: Tuberculosis
TGF: Transforming growth factor
TSL: Thermosensitive liposome
TSTn: Tuberculin skin test negative
XDR: Extensive drug resistance
β -CD: β -Cyclodextrins
